# Effects of environmental tobacco smoke exposure on brain functioning in never‐smoking adolescents

**DOI:** 10.1002/brb3.1619

**Published:** 2020-07-01

**Authors:** Joyce Dieleman, Marloes Kleinjan, Roy Otten, Hein T. van Schie, Vivian Heuvelmans, Maartje Luijten

**Affiliations:** ^1^ Department of Epidemiology and Research Support Trimbos Institute Utrecht The Netherlands; ^2^ Behavioural Science Institute Radboud University Nijmegen The Netherlands; ^3^ Pluryn Nijmegen the Netherlands; ^4^ Arizona State University Tempe AZ USA; ^5^ Interdisciplinary Social Sciences Utrecht University Utrecht The Netherlands; ^6^ Department of Cognitive Neuroscience Radboud University Medical Center Nijmegen The Netherlands

**Keywords:** environmental tobacco smoke (ETS) exposure, ERPs, nicotine dependence

## Abstract

**Introduction:**

Brain functioning, as indexed by event‐related potentials (ERPs) representing smoking cue reactivity, inhibitory control, and reward processing, has been found to be compromised in smokers. However, whether environmental tobacco smoke (ETS) exposure in never smokers results in similar brain changes is unknown. This question is particularly relevant during adolescence, given ongoing brain maturation and a high risk of smoking initiation. The present study tested the associations between ETS exposure and ERPs reflecting cue reactivity (P3, LPP), inhibitory control (N2, P3), and reward processing (anticipation P3 (P3), feedback‐related negativity (FRN)) among never‐smoking adolescents.

**Methods:**

Eighty‐four never‐smoking adolescents (nonexposed = 32, exposed = 52) performed a smoking cue reactivity, a Go/NoGo, and a monetary incentive delay (MID) task while ERPs were measured.

**Results:**

Exposed and nonexposed groups did not differ in ERPs reflecting smoking cue reactivity, inhibitory control, and reward processing. A negative correlation between ETS exposure and the anticipatory P3 suggests reduced anticipatory reward sensitivity for nondrug rewards with increased levels of ETS exposure. However, since this effect was not consistent across analyses, no strong conclusions can be formulated. In the current study, few participants reported high levels of ETS exposure; therefore, further study is necessary.

**Conclusions:**

Nevertheless, from this study, it can be concluded that low‐to‐moderate exposure to ETS during adolescence does not result in functional brain changes related to smoking cue reactivity, inhibitory control, and reward processing.

## Graphical abstract

This study tested the associations between ETS exposure and ERPs reflecting cue reactivity (P3, LPP), inhibitory control (N2, P3), and reward processing (anticipation P3 (P3), feedback‐related negativity (FRN)) among never‐smoking adolescents.

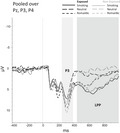

## 1 INTRODUCTION

Environmental tobacco smoke (ETS) exposure is one of the most common health hazards in society. It is estimated that 40% of the world's youths are exposed to ETS (Öberg, Jaakkola, Woodward, Peruga, & Prüss‐Ustün, 2011). Smoking in the environment of the adolescent increases the risk of smoking initiation and dependence during adolescence (de Leeuw, Engels, Vermulst, & Scholte, 2009; Kleinjan et al., 2009), but little is known about the underlying neurobiological mechanisms at work. The aim of this study was to examine whether ETS exposure during adolescence affects brain functioning linked to motivational processes and behavioral control in never‐smoking adolescents.

Evidence suggests that particularly adolescents are vulnerable to the effects of ETS exposure. Studies have shown higher levels of nicotine and cotinine in urine in children than in adults after equal exposure to ETS (Willers, Skarping, Dalene, & Skerfving, 1995), indicating that youths might absorb more nicotine. In line with this finding, it has been shown that preadolescent rats absorb more nicotine as a result of ETS exposure as compared to adult rats and that preadolescent tobacco smoke exposure increases the risk of nicotine dependence in the future (Yamada et al., 2010). It has also been shown in adolescent rats that exposure to ETS throughout adolescent neurodevelopment alters the cholinergic system in brain regions associated with nicotine dependence, suggesting that the biological mechanisms underlying nicotine dependence can be activated by ETS exposure throughout adolescence (Abreu‐Villaça et al., 2003). Additionally, the adolescent brain is still developing; hence, the brain's motivational system and behavioral control and inhibition system are still not fully developed (Arain et al., 2013; Casey, Getz, & Galvan, 2008).

Furthermore, several studies found an association between ETS exposure and the occurrence of behavioral symptoms of nicotine dependence among never‐smoking youths, such as craving, cue‐triggered wanting to smoke, irritability, and trouble concentrating (Bélanger et al., 2008; Okoli et al., 2016; Schuck, Kleinjan, Otten, Engels, & Difranza, 2013). The positive association between craving, cue‐triggered wanting to smoke, and ETS exposure prompted the idea that ETS exposure may result in functional brain changes related to smoking cue reactivity. Besides, the possibility exists that ETS exposure during adolescence results in functional brain changes related to response inhibition and reward processing. The idea that ETS exposure during adolescence may result in functional brain changes related to response inhibition and reward processing stems from studies focused on the effects of intrauterine cigarette smoke exposure. Most of these studies show that children who were prenatally exposed to cigarette smoke were more likely to show aberrant brain functioning related to response inhibition and reward processing during adolescence (Bennett et al., 2009; Boucher et al., 2014; Holz et al., 2014; Longo, Fried, Cameron, & Smith, 2013; Müller et al., 2013). In a study on the acute effects of ETS exposure on the neurobiological system in adults, Brody et al. (2011) exposed participants to ETS for 1 hr during which the participants sat in the passenger's seat of a car. Using positron emission tomography (PET), they showed that nicotine inhaled from ETS exposure crossed the blood–brain barrier, resulting in the occupation of nicotinic acetylcholine receptors (nAChRs) in motivational and inhibition‐related brain regions of adult smokers and nonsmokers. Prior research thus has primarily focused on prenatal cigarette smoke exposure and adult exposure, but to our knowledge ETS exposure has not been related to alterations in brain functioning during adolescence. The above studies suggest the possibility that ETS exposure during adolescence may be associated with functional brain changes related to cue reactivity, reward processing, and response inhibition.

One approach to study functional brain changes in the laboratory is through the use of electroencephalography (EEG) and event‐related potentials (ERPs). With EEG, neuronal activity is measured with electrodes at the surface of the scalp. Within the EEG signal, several positive and negative brain waves can be identified. These potentials are called ERPs when time‐locked to a discrete event or defined stimulus. Over the years, a lot of ERP research has been done and a variety of ERP components have been identified relating to cue reactivity, reward processing, and response inhibition.

While the effects of ETS exposure on brain functioning in adolescents have not yet been investigated, the functional mechanisms underlying addictive behaviors have been studied extensively. The current study, therefore, investigated the possibility that ETS exposure may affect brain mechanisms that have been implicated in addiction. In particular, addiction is suggested to be characterized by an imbalance between motivational processes and behavioral control and inhibition processes (Field & Cox, 2008; Goldstein & Volkow, 2011). EEG studies comparing substance‐dependent individuals against controls have consistently found addictive behaviors to be reflected in altered ERPs, indicating increased motivated attention to substance‐related cues as well as decreased inhibitory control (Littel, Euser, Munafò, & Franken, 2012; Luijten et al., 2014). In the current study, we investigated whether ETS also affects ERP components associated with motivation/reward and behavioral control and inhibition.

One way of studying motivational processes is by focusing on cue reactivity. The P3 and late positive potential (LPP) are ERPs associated with cue reactivity. Both the P3 (300–500 ms, medial central and parietal) and the LPP (300–700 ms, centroparietal) reflect attentional processing of salient stimuli as well as the continuation of attentional processing to facilitate memory storage (Cuthbert, Schupp, Bradley, Birbaumer, & Lang, 2000; Hajcak & Olvet, 2008; Koenig & Mecklinger, 2008; Littel et al., 2012; Polich, 2007). A meta‐analysis studying the neural basis of smoking‐related cue reactivity using EEG found enlarged P3 and LPP amplitudes for cigarette relative to neutral cues in smokers but not in controls, probably indicating increased motivated attention to smoking cues in smokers (Littel et al., 2012).

Another way to study motivational processes is by looking into reward processing of nondrug rewards. The neural basis of reward processing in addictive behaviors has often been studied by measuring brain activity in response to mostly monetary rewards. Reward processing can be divided into two phases, with reward anticipation and reward outcome reflecting distinct processes (Broyd et al., 2012). The P3 for anticipation (P3) and the feedback‐related negativity for the outcome (FRN) are ERPs associated with reward processing. The P3 (350–600 ms, centroparietal) reflects the allocation of attention toward reward‐predicting stimuli (Broyd et al., 2012; Pfabigan et al., 2014), resulting in reward‐seeking behavior. The FRN (200–300 ms, frontocentral) reflects the response to negative feedback or worse than expected outcomes (Broyd et al., 2012; Yaple, Shestakova, & Klucharev, 2018). While ERP studies related to reward anticipation in smokers are lacking, several fMRI studies have observed reduced anticipatory brain activation for monetary rewards in smokers (Fedota et al., 2015; Rose et al., 2013; Sweitzer et al., 2016; van Hell et al., 2010). In addition, an ERP study related to reward anticipation found a decreased P3 amplitude in cocaine users versus controls (Goldstein et al., 2008). These results indicate less sensitivity to potential monetary rewards in addicted individuals. Regarding reward outcome, the reinforcement learning theory posits that worse than expected outcomes, reflected as an increase in the negativity of the FRN, co‐occur with a decrease in the activity of the midbrain dopamine neurons (Holroyd & Coles, 2002). Parvaz et al. (2015) reported deficits in reinforcement learning in cocaine‐addicted individuals, as indexed by an absence of FRN amplitude modulation in this group. Moreover, neuroimaging studies in smokers also found reduced brain activation in response to non–drug‐related reward outcome (Baker et al., 2017; Schuck, Otten, Engels, & Kleinjan, 2012; Wilson et al., 2014). These studies point in the direction of aberrant reward processing in substance‐dependent individuals relative to controls.

In addition to motivational and reward processes, the importance of behavior control and inhibition processes in addictive behaviors has also been emphasized. The N2 and P3 amplitudes are ERPs associated with inhibitory control. The N2 (250–350 ms, frontocentral) reflects early detection of conflict, whereas the P3 occurs later during the process of inhibition, reflecting actual inhibition of the motor system (Buzzell, Fedota, Roberts, & McDonald, 2014; Groom & Cragg, 2015) It has been shown that substance‐dependent individuals, including smokers, have more difficulties inhibiting their responses (Smith, Mattick, Jamadar, & Iredale, 2014). In a similar vein, a systematic review of studies focusing on inhibitory control in addiction, including smoking, revealed decreased N2 amplitudes associated with conflict detection in smokers compared with controls (Luijten et al., 2014). Additionally, some studies have related prenatal cigarette smoke exposure to changes in brain functioning during adolescence (Bennett et al., 2013; Boucher et al., 2014; Holz et al., 2014; Longo et al., 2013). More specifically, Boucher et al. (2014) investigated the effects of prenatal cigarette smoke exposure on inhibitory control and found reduced N2 and P3 components, similar to the neurobiological changes related to smoking (Luijten et al., 2014). This suggests that ETS exposure during adolescence may also result in functional brain changes.

The current study is the first to examine the effects of ETS exposure on brain functioning in never‐smoking adolescents by assessing cue reactivity, reward processing, and inhibitory control using ERPs in exposed and nonexposed adolescents. Using a smoking cue‐reactivity paradigm, we expected smoking‐related cue reactivity (i.e., more attention toward smoking‐related cues) to be reflected in enlarged P3 and LPP components in exposed compared with nonexposed adolescents. Using a monetary incentive delay task, we expected less sensitivity to monetary rewards, as reflected in a reduced anticipatory P3 amplitude, in exposed compared with nonexposed adolescents. For reward outcome, we expected a decrease in the negativity of the FRN component in exposed compared with nonexposed adolescents reflecting deficits in reinforcement learning. Using a Go/NoGo task, we expected N2 and P3 components to decrease in response to NoGo stimuli in exposed compared with nonexposed adolescents, which would indicate that exposed individuals have more difficulties to inhibit their response. In addition to differences between exposed and nonexposed adolescents, we also expected dose‐dependent ETS effects.

## 2 MATERIALS AND METHODS

### 2.1 Participants and procedures

Eighty‐four never‐smoking adolescents participated in this study divided into a nonexposed and exposed group (Table 1). Participants were excluded when they (a) smoked more than a single puff of a cigarette once, (b) had a serious head injury, or (c) used psychoactive medication. Participants received 50 euros in gift vouchers after study completion. Both participants and their parents provided informed consent, and the Medical Ethical Committee of Arnhem‐Nijmegen approved the study protocol (#2015‐2120). Participants were invited to the Behavioural Science Institute (BSI) Lab at the Radboud University for the test session. Before EEG data acquisition, participants and one of the parents were asked to fill in several questionnaires. Basic information such as educational level, age, and gender was measured in addition to self‐report measures of current ETS exposure, pubertal development for the adolescents and familial risk of smoking, and whether the mother smoked during pregnancy for one of the parents. Given that some mothers smoked during pregnancy in either the exposed group (21% of moms who smoked during pregnancy) or nonexposed group (6% of moms who smoked during pregnancy), we added smoking during pregnancy as a covariate in all analyses. Familial nicotine dependence risk, gender, and pubertal development were also included as covariates in all analyses.

**TABLE 1:** Demographics

	Nonexposed (*N* = 32)			Exposed (*N* = 52)				
	Mean	*SD*	Range	Mean	*SD*	Range	t/X2	*p*
Gender (% male)	81%			64%			2.999	.083
Education							0.118	.731
% low education	66%			69%				
% high education	34%			31%				
Age	13.84	0.77	13–16	14.37	1.09	13–17	−2.576	**.012** *
ETS exposure	0			8.48	7.11	1–41	−8.597	**.000** ***
PDS score	2.91	0.60	1.4–3.6	3.03	0.62	1.4–4.0	−0.929	.365
Familial risk^a^	1.15	1.94	0–7.5	2.48	2.13	0–9.0	−2.850	**.006** *
Smoking during pregnancy (% yes)^a^	6%			21%			3.303	.069

Statistically significant *p*‐values are indicated in bold.

Abbreviations: ETS, environmental tobacco smoke, PDS, pubertal development scale.

^a^ *N* = 31 for the nonexposed participants instead of *N* = 32 and *N* = 51 for the exposed participants instead of 52 due to two missings on these variables.

* p <0.05

*** p <0.001

### 2.2 Questionnaires—ETS exposure measure

Participants were asked to report on the frequency of ETS exposure in their environment: “How often does your ‘father’ smoke when you are around?” Response items ranged from (0) my “father’ smokes, but not when I am around to (8) more than five times a day.” Participants filled out this question for relatives (father, mother, siblings), friends (best friend, friends in general), and others in their environment. Participants’ responses were combined to establish a sum score for ETS exposure, with a range from 0 to 48, where a higher score indicates more exposure. Previous research has indicated that children are reliable reporters of the smoking behavior in their social environment (Harakeh, Engels, Vries, & Scholte, 2006). We cross‐validated the adolescent report of parental smoking with the self‐report of the parents (father and mother) on their own smoking status, and we found that in 84.2% of the cases the child correctly reported whether their father was smoking or not. In 91.5% of the cases, the child correctly reported whether their mother was smoking or not. This indicates that the self‐report of the adolescents on ETS exposure in their environment was quite reliable.

*Pubertal Development Scale*. Participants filled out the Pubertal Development Scale (PDS) (Petersen, Crockett, Richards, & Boxer, 1988), a self‐report questionnaire containing questions on secondary sexual characteristics. For detailed information, see supplementary materials.

### 2.3 Familial risk

To obtain an estimate of participants’ familial vulnerability to develop nicotine dependence, a familial risk score was created. The questionnaire, completed by one of the parents, addressed three domains: (a) their current smoking behavior and frequency, (b) level of nicotine dependence for the period in which they smoked the heaviest (could either be now or in the past), and (c) smoking behavior of their parents (i.e., grandparents of participants). The scores from the three domains were summed for both parents, resulting in two total scores (father and mother). Subsequently, the two total scores for both parents were summed and averaged. Detailed information on the calculation of this score is available in the supplementary materials.

### 2.4 Smoking during pregnancy

To assess smoking of the mother during pregnancy, parents were asked the following: “did you(r wife) smoke during the pregnancy of your son/daughter?” Response options were yes (1) and no (0).

### 2.5 Experimental tasks

For all experimental tasks, we used Presentation software (version 21.0; Neurobehavioral Systems, Inc., www.neurobs.com). Before the start of the tasks, we thoroughly explained the tasks.

### 2.6 Smoking cue‐reactivity task

A total of 32 neutral pictures, 32 smoking pictures, and 32 romantic pictures were presented to participants for passive viewing. By adding the romantic category, we can investigate whether attentional processing is increased in general for rewarding cues (romantic pictures as well as smoking pictures) or whether the increase in attentional processing is smoking‐specific as a result of ETS exposure. Smoking pictures showed people smoking or holding smoking‐related objects. Neutral and romantic pictures showed people in similar scenes, however, without smoking or holding smoking‐related objects. Smoking‐related, neutral and romantic pictures were matched for number and gender of the persons displayed as well as colors of the pictures. Pictures were presented for 1,000 ms, followed by an interstimulus interval between 800 ms and 1,200 ms. All pictures were presented once, resulting in 96 trials. Pictures were presented one at a time, and the order of the trial type was randomized such that no more than three pictures of the same stimulus category were displayed in a row.

### 2.7 Monetary Incentive Delay (MID) task

Reward processing was measured with the MID task (Broyd et al., 2012). Each trial started with a cue presentation (500 ms). The color of the cue (blue or yellow, counterbalanced across participants) indicated whether one can either win money (50 cents) or receive no money (0 cents). After the cue presentation, a fixation cross was presented (100–1,400 ms), followed by the target presentation (white star). Participants were instructed to press the button box as fast as possible upon presentation of the target. If participants responded within an individually determined time window, they won. The time window was individually adapted depending on their performance (following correct trials, the response time for that cue decreased by 10 ms, while following incorrect trials, it increased by 20 ms), aiming to achieve a hit rate of approximately 66%. The length of the time window at the start of the experiment was based on a total of 8 practice trials. After the target presentation, another fixation cross was presented (800–1,200 ms), followed by the presentation of feedback (1,000 ms) indicating whether the participant was fast enough (√) (+ 50 cents (in case of rewarding trials) or not (X) and their cumulative gain (total amount of money gained so far)). In total, 60 sixty rewarding and 60 nonrewarding stimuli were presented in a randomized order. The total gain on this task reflected the total reimbursement that participants received afterward.

### 2.8 Go/NoGo task

**FIGURE 1:**
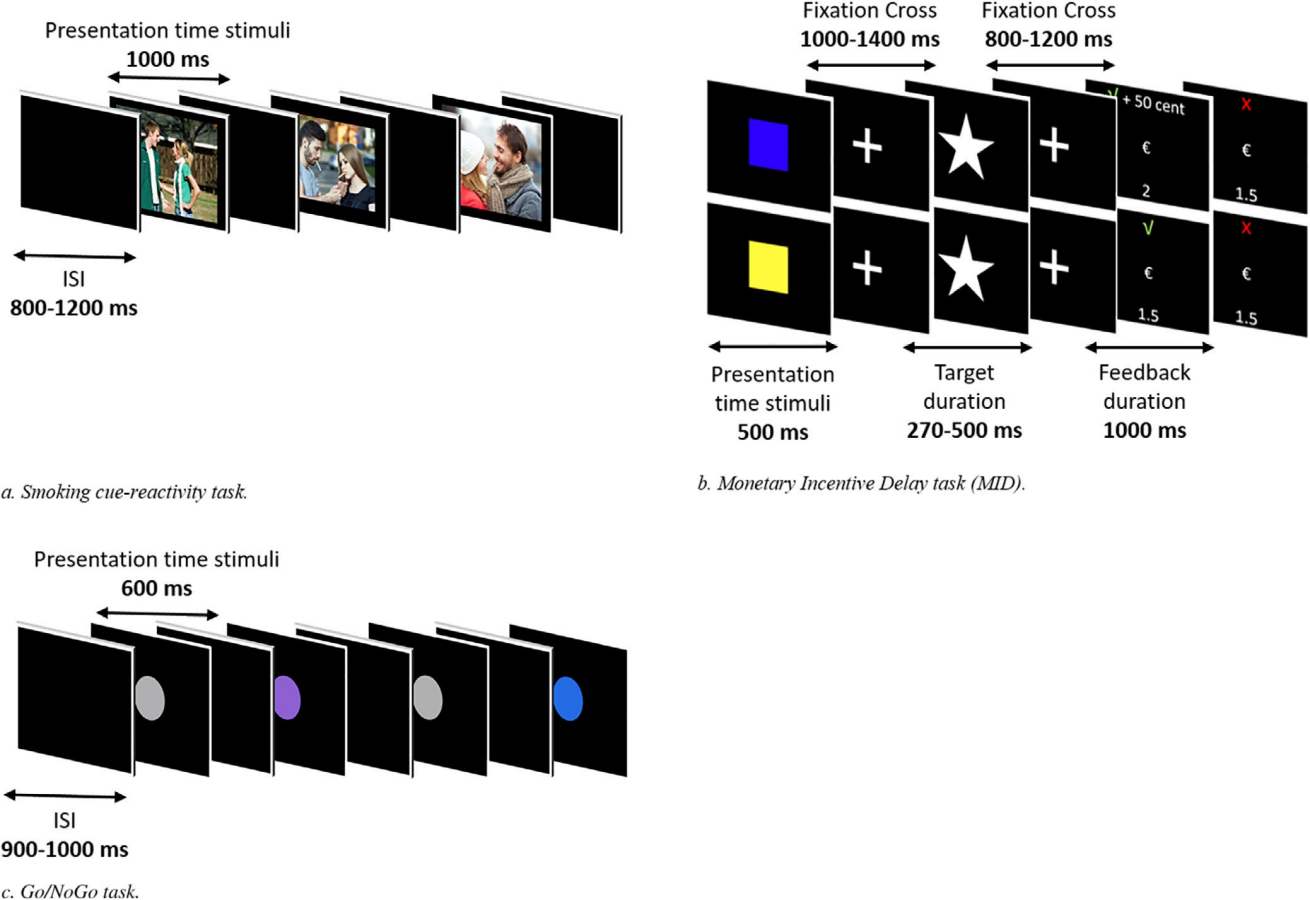
Experimental tasks

Inhibitory control was assessed with a Go/NoGo task using colored circles as stimuli. Colors of the circles indicated whether it was a Go (grey, 65%, *N* = 249), IfGo (purple, 17.5%, *N* = 67), or NoGo (blue, 17.5%, *N* = 67) trial. Participants were instructed to press the button box as fast as possible upon presentation of Go and IfGo trials and withhold their response in NoGo trials. Each circle was displayed for 600 ms, followed by a black screen (900–1,000 ms). See Figure 1 (i.e., cue reactivity in Figure 1a, MID in Figure 1b, Go/IfGo/NoGo in Figure 1c) for an overview of the experimental tasks.

### 2.9 Cotinine measurement

To biochemically verify ETS exposure, saliva samples of participants were collected to measure the levels of cotinine, a metabolite of nicotine. Cotinine levels were analyzed using liquid chromatography coupled with mass spectrometry, with a quantification limit of < 1.0 µg/L. Range of cotinine levels varied from 0 to 4.7 µg/L (*M* = 0.53, *SD* = 1.06). Two participants had no cotinine values because too little saliva was collected. A dichotomous cotinine measure was created with no cotinine detected (0) versus. cotinine detected (1). The dichotomous cotinine measure was compared with the dichotomous ETS exposure measure using a chi‐square test to test whether cotinine in saliva was associated with the self‐reported ETS exposure measure. Chi‐square values showed that cotinine was significantly associated with the ETS exposure measure, *χ* ^2^(1) = 6.131*, p* = .013, indicating an overlap between the two variables. Cotinine measures in saliva capture ETS exposure in the previous 1–3 days (Racicot, McGrath, & O’Loughlin, 2011). Cotinine was detected in 37.3% of participants who self‐reported ETS exposure, whereas in 62.7% of the participants who reported exposure to ETS, no cotinine was detected. In 90.3% of participants who self‐reported no ETS exposure, no cotinine was detected, whereas in 9.7% of the cases, cotinine was found. Nonperfect overlap between saliva cotinine measures and self‐reported ETS exposure may result from the fact that saliva cotinine only captures ETS exposure in the previous 72 hr. Assignment to the nonexposed or exposed group was based on the self‐report measures. The cotinine measures were used to validate the self‐report measures.

### 2.10 Valence and Arousal ratings

Subjects rated half of the pictures of all the different stimulus categories (neutral, smoke, romantic) in terms of valence (from negative (−100) to positive (+100)) and arousal (from not arousing to highly arousing) using visual analogue scales to test how participants perceived the images.

### 2.11 Electrophysiological recording and offline data processing

Details about EEG recording and offline data processing are included in supplementary materials.

### 2.12 EEG segmentation per task per stimulus type

For an overview of the time window of segmentation, the time interval selected for each ERP component, selected electrodes, analyzable segments (mean and range) per stimulus type/condition, and the number of participants excluded from each task; see Table 2. Detailed information and references are included in supplementary materials.

**TABLE 2:** EEG offline data processing related to segmentation

Task paradigm	Segmentation	ERP interval	Electrode selection	Analyzable segments (mean and range)	No. of participants excluded for analyses because of too many artifacts
Cue reactivity	1,400 ms (−400–1,000 ms)	**P3** (250–400 ms)	P3, Pz, P4	**Smoking pictures:** 30 (range: 22–32)	3
		**LPP** (450–1,000 ms)	P3, Pz, P4	**Neutral pictures:** 31 (range: 20–32)	
				**Romantic pictures:** 31 (range: 24–32)	
Monetary incentive delay *Anticipation*	1,900 ms (−400–1,500 ms)	**P3** (275–500 ms)	Pz, P3, P4, CP1, CP2	**Reward:** 57 (range: 46–60)	2
				**Nonreward:** 58 (range: 45–60)	
Monetary incentive delay *Outcome*	2,000 ms (−400–1,600 ms)	**FRN** (200–300 ms)	FCz, Fz, FC1, FC2	**Reward correct:** 40 (range: 30–49)	7
				**Reward incorrect:** 16 (range: 10–22)	
Go/IfGo/NoGo	1,200 ms (−400–800 ms)	N2 (200–320 ms)	F3, F4, Fz, FCz	**Go:** 231 (range: 120–249)	2
		P3 (320–500 ms)	FC1, FCz, Cz, FC2	**IfGo:** 62 (range: 28–67)	
				**NoGo:** 51 (range: 29–65)	

Note

Overview of segmentation per task, the time interval selected for each ERP component, electrode selection, analysable segments (mean and range) per stimulus type and the number of participants excluded for analysis. ERP = event related potential.

### 2.13 Analyses

Analyses were conducted in three steps. First, relevant ERPs of nonexposed and exposed individuals were compared across task conditions and Group × Condition interactions. Second, correlational analyses with ETS exposure and ERP difference scores (for ERP difference scores, see Table 3) were applied in the total and subsample. Third, hierarchical regression analyses were applied within the exposed group to test the dose–response relationship between ETS exposure and ERP difference scores. Gender, pubertal development, familial risk, and smoking during pregnancy were included as covariates in all, except bivariate correlational, analyses. By default, we report the results of the analyses with covariates in the main text as the confirmatory analysis as well as the results of the bivariate correlational analysis. More exploratory, and as a sensitivity check, we also performed the analyses without covariates and report the outcomes of these analyses in the main text if the inclusion of covariates changed the significance or direction of the effects of primary interest.

**TABLE 3:** Difference scores for correlational and regression analysis

Task paradigm	Difference scores per event‐related potential	Conceptual measure
Cue reactivity	P3 Smoking minus Neutral LPP Smoking minus Neutral	Early motivated attention Late motivated attention
MID—anticipation MID—outcome	P3 Reward minus Nonreward FRN Reward_Incorrect minus Reward_Correct	Anticipatory reward sensitivity Negative reward prediction error
Go/IfGo/NoGo	N2 NoGo minus Go P3 NoGo minus Go	Conflict detection Actual inhibition

Note

Overview of the calculated difference scores per ERP. All difference scores were averaged over included electrodes for each component.

More specifically, in the first step, RM‐ANCOVAs (with Greenhouse–Geisser‐adjusted *p*‐values if needed) were applied to compare individuals exposed to ETS with individuals who were not exposed to ETS. The between‐subjects factor in all RM‐ANCOVAs was Group (nonexposed individuals vs. exposed individuals). The within‐subjects factor for the cue‐reactivity task was Picture Type (smoke vs. neutral vs. romantic). The within‐subjects factor for the anticipatory phase of the MID task was Reward (reward vs. nonreward), and the within‐subjects factor for the outcome phase of the MID task was Reward_Outcome (correct vs. incorrect). The within‐subjects factor for the Go/NoGo task was Inhibition with three levels (Go vs. IfGo vs. NoGo). For all ERP analyses, the electrode was included as an additional within‐subjects factor (for selected electrodes, see Table 2). For behavioral analyses, a Group × Picture type RM‐ANCOVA was performed to analyze differences in valence and arousal ratings for the pictures of the cue‐reactivity task. A Group × Reward RM‐ANCOVA was performed to analyze reaction times (RTs) in the MID task and a Group × Inhibition RM‐ANCOVA to analyze behavioral NoGo accuracy in the Go/NoGo task. In all analyses, follow‐up tests were carried out to test differences between groups, task conditions, and Group × Condition interactions involving pairwise comparisons between estimated marginal means. Additionally, the Bonferroni adjustment was used to correct for multiple comparisons in the follow‐up analyses.

In the second step, correlational analyses were conducted to first determine significant bivariate relationships between covariates, dependent variables, and independent variables. Specifically, correlations were computed between the predictors and covariates (ETS exposure, gender, familial risk, pubertal status, and smoking during pregnancy) as well as among the predictors and the created difference scores per task for all ERP components. In addition, bivariate correlational analyses were conducted between ETS exposure and the valence and arousal ratings as well as between the valence and arousal ratings and the P3 and LPP components of the cue‐reactivity task.

In the third step, hierarchical regression analyses were performed within the group of exposed individuals to assess the dose–response relationships between brain functioning and ETS exposure with the previously created difference scores per ERP component as outcome variables and ETS exposure as the independent variable. All covariates were included in the first step and ETS exposure in the second step.

All analyses with and without covariates are reported in the supplementary materials, Tables S1–S10 for the cue‐reactivity task, Tables S11–S14 for the valence and arousal ratings related to the cue‐reactivity task, Tables S15–S24 for the MID task, and Tables S25–S37 for the Go/NoGo task. Correlation matrices between ETS exposure and covariates (Table S38) and between ETS exposure, covariates, and ERP difference scores (Table S39) as well as correlation matrices between ETS exposure, P3 and LPP components, and valence and arousal ratings (Table S40) are also reported in supplementary materials. Correlations between ETS exposure, ERPs, and valence and arousal ratings are discussed in the main text as well, and other correlations (i.e., between covariates and ERPs) are reported in the supplementary materials.

## 3 RESULTS

**FIGURE 2:**
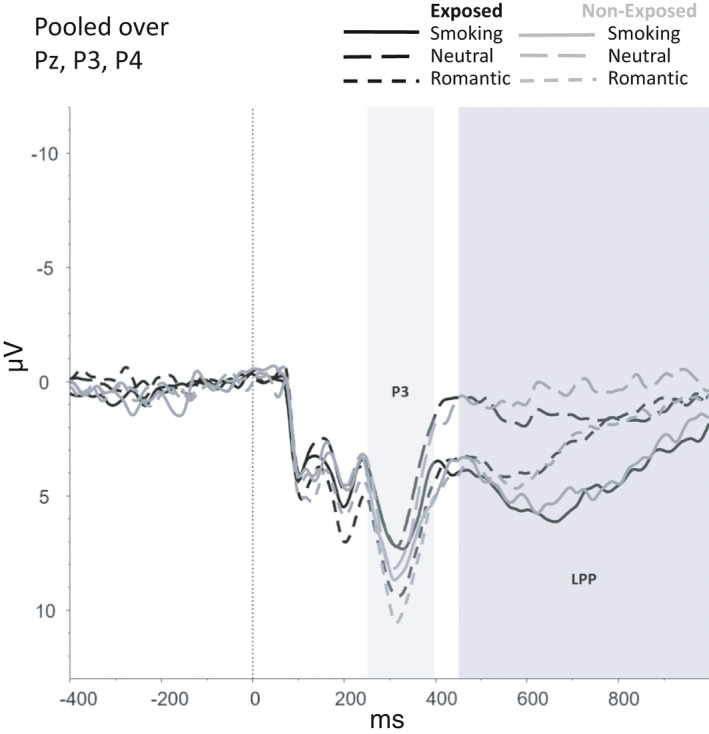
Smoking cue‐reactivity, P3 and late positive potential

**FIGURE 3:**
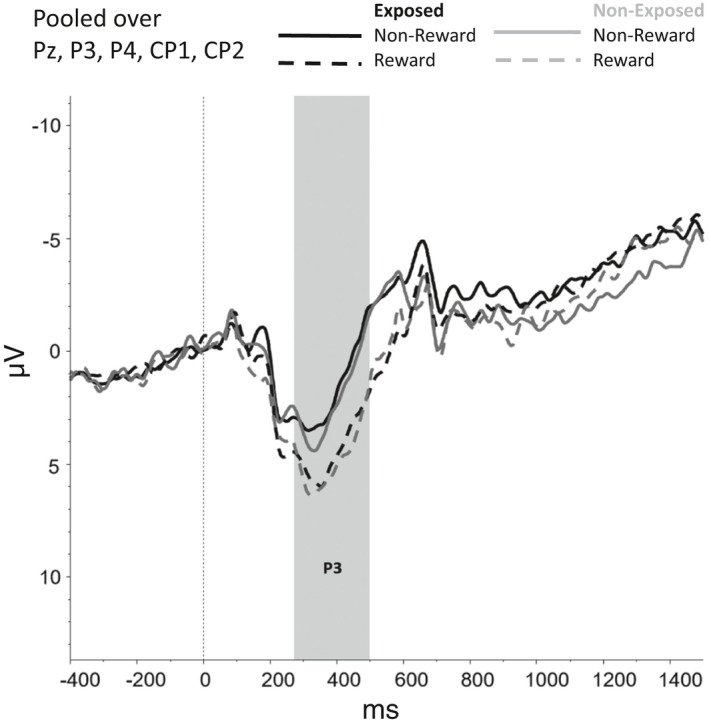
Reward processing—anticipation, P3

**FIGURE 4:**
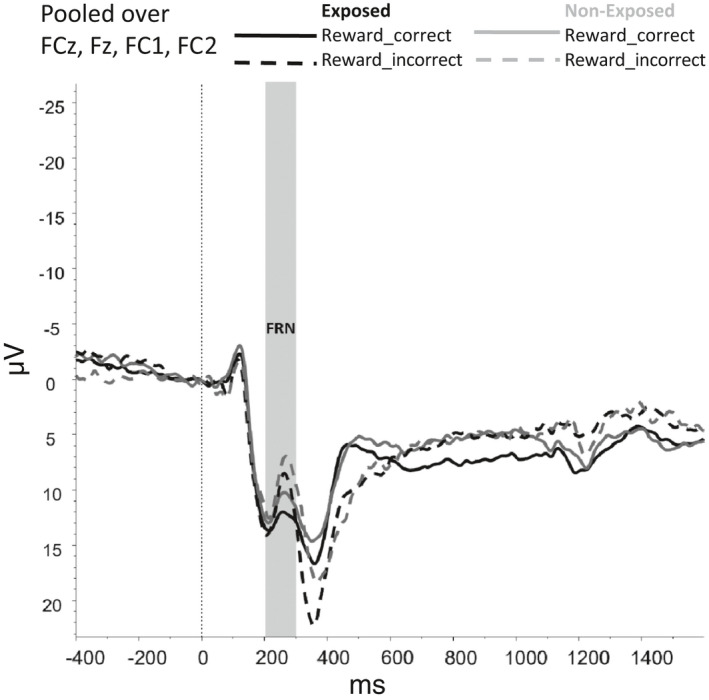
Reward processing—outcome, feedback‐related negativity

**FIGURE 5:**
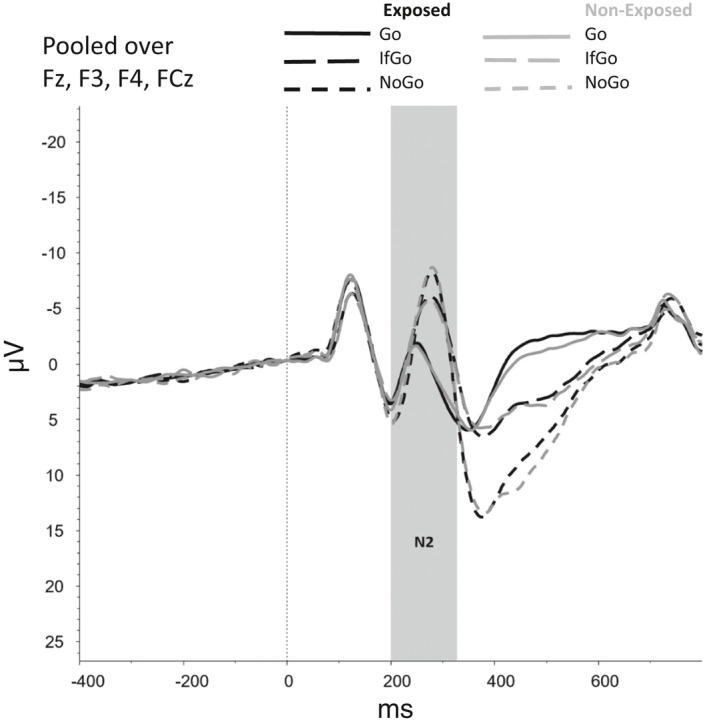
Inhibitory control—Go/NoGo task, N2

**FIGURE 6:**
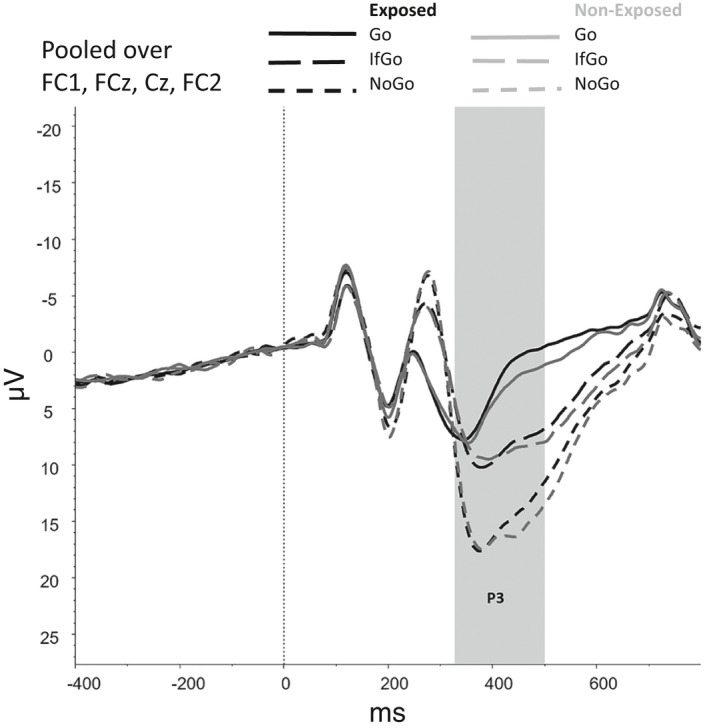
Inhibitory control—Go/NoGo task, P3

Figures 2, 3, 4, 5, 6 show the amplitudes of all ERPs of interest related to cue reactivity (P3 and LPP) (2), reward processing anticipation (P3) (3), reward processing outcome (FRN) (4), and inhibitory control (N2) (5) and inhibitory control (P3) (6) for the different conditions in both groups.

### 3.1 Nonexposed versus. exposed individuals

*Cue reactivity*

A main effect of Picture Type for the valence ratings was found (*F*(1,28) = 604.99, *p* < .001). No main effect of arousal was observed (*F*(1,65) = 10,12, *p* = .549). For the valence ratings, the smoking pictures were most negatively rated. The valence ratings for the smoking pictures differed significantly from the neutral (*p* < .001) and romantic pictures (*p* < .001). The valence ratings for the romantic pictures differed significantly from the neutral pictures as well (*p* < .001). This indicates that smoking pictures were most negatively rated, followed by neutral and romantic pictures, which were positively rated. No interactions effects including Group were found for both the valence and arousal ratings. See Table S11–S14 for the statistical values. From this, we can conclude that never‐smoking participants perceive smoking pictures as negative.

For the P3, a main effect was found for Picture type, *F*(2, 146) = 3.62, *p* = .029, indicating that P3 amplitudes were higher for romantic than for smoking (*p = *.002) and neutral pictures (*p* < .001). No significant Group effect (*F*(1,73) = 0.23, *p* = .630) or interactions that included Group (*F*(2,146) = 0.05, *p* = .952) were found for the P3.

For the LPP, a main effect was found for Picture Type (*F*(92, 146) = 5.37, *p* = .006) showing that the LPP amplitudes were higher for smoking than for romantic (*p < *.001) and neutral pictures (*p < *.001). Additionally, LPP amplitudes were higher for romantic pictures compared with neutral pictures (*p = *.002). No other main effects (*F*(1,73 = 0.19, *p* = .663) or interaction effects that included Group (*F*(2,146) = 0.51, *p* = .603) were found for the LPP.

#### 3.1.1 Reward processing

No effect of Group, Trial Type (reward vs. nonreward), or interaction between Group and Trial Type on reaction times was found. However, the model without covariates showed a main effect of Trial Type (*F*(1, 82) = 44.23, *p* < .001), indicating that overall, participants responded faster during rewarding compared with nonrewarding trials.

For the anticipatory P3, a main effect was found for Trial Type, *F*(1, 74) = 9.68, *p* = .003, indicating that P3 amplitudes were larger for rewarding compared with nonrewarding trials (*p* < .001). The main effects of Group (*F*(1, 74) = 0.26, *p* = .610) and interaction effects involving Group (*F*(1, 74) = 0.46, *p* = .502) were nonsignificant.

For the FRN, no main effects were observed for Group (*F*(1,69) = 3,502, *p* = .066) and Reward_Outcome (*F*(1,69) = 1.779, *p* = .187) and neither interaction effects between Group and Reward_Outcome (*F*(1,69) = 0.407, *p* = .525). However, the model without covariates did show a main effect of Reward_Outcome *F*(1, 75) = 6.087, *p* = .016, indicating that FRN amplitudes were larger (i.e., more negative) for Reward_Incorrect than for Reward_Correct trials, reflecting the difference between the omission of gains and receiving gains.

#### 3.1.2 Inhibitory control

A main effect of Trial Type (Go, NoGo, IfGo) on accuracy was found, *F*(1.02, 76.43) = 9.64, *p* = .003. Accuracy was lower on NoGo trials compared with both Go trials (*p* < .001) and IfGo trials (*p* < .001), indicating that participants made more errors in NoGo trials than in Go and IfGo trials. Accuracy did not differ between Go and IfGo trials (*p* > .999). No main effect (*F*(1,75) = 3.68, *p* = .059) or interaction effect that included Group (*F*(1.02,76.43) = 2.89, *p* = .093) was found.

For the N2 component, no main effects were observed for Group (*F*(1,75) = 0.53, *p* = .468) and Trial Type (*F*(1.75, 131.27) = 1.76, *p* = .180) and neither an interaction effect between Group and Trial Type (*F*(1.75, 131.27) = 1.75, *p* = .183). However, the model without covariates showed a main effect of Trial Type (*F*(1.71, 138.75) = 61.39, *p* < .001), indicating that N2 amplitudes were smaller for Go trials than for NoGo and IfGo trials (both *p*s < 0.001).

For the P3, a main effect was found for Trial Type, *F*(1.58, 118.45) = 21.94, *p* < .001, showing that mean amplitudes differed significantly between each of the Trial Types (all *p*s < 0.001), with amplitudes being highest for NoGo trials, followed by IfGo trials and Go trials. No main effect of Group (*F*(1,75) = 0.09, *p* = .764) nor an interaction effect between Group and Trial Type (*F*(1.58, 118.45) = 0.69, *p* = .741) was observed.

### 3.2 Correlational analyses

Correlational analyses within the exposed group showed a significant negative correlation between ETS exposure and the anticipatory P3 difference score reflecting anticipatory reward sensitivity (*r*(49) = −0.29, *p* = .042) indicating reduced anticipatory reward sensitivity for non–drug‐related rewards in participants with more ETS exposure; see Figure S1 for a scatterplot of this correlation. All other correlations between ETS exposure and ERPs were nonsignificant in both the exposed group and nonexposed group.

The bivariate correlational analysis between the valence and arousal ratings and ETS exposure across all participants was only significant for the valence ratings of the smoking‐related pictures (*r*(83) = .234, *p* = .032). This correlation indicates that the higher the exposure, the more positive (i.e., less negative) the smoking pictures were perceived. All other correlations between the valence and arousal ratings and the P3 and LPP components were nonsignificant.

### 3.3 Regression analyses within the exposed group

#### 3.3.1 Cue reactivity

The step 1 regression models with the P3 and LPP components of the cue‐reactivity task were not significant (*p* = .317, *p* = .790, respectively). ETS exposure did not explain additional variance in the P3 (*∆R* ^2^ *= *.001, *p = *.833) and LPP components (*∆R* ^2^ = .006, *p = *.580).

#### 3.3.2 Reward processing

The step 1 regression model with the P3 component of the MID task was not significant (*p* = .192). ETS exposure did not explain additional variance in the P3 component (*∆R* ^2^ = .04, *p* = .152). For the FRN, significant regression equations were found for both step 1 (*p* = .005) and step 2 (*p* = .006), with gender as the significant predictor (step 1: *β*_gender_ = −0.53, *t*(4,41) = −3.28, *p* = .002, step 2: *β*_gender_ = −0.55, *t*(5,40) = −3.42, *p* = .001). ETS exposure did not explain additional variance in the FRN component (*∆R* ^2^ = .03, *p* = .203).

#### 3.3.3 Inhibitory control

The step 1 regression models for the N2 and P3 components of the Go/NoGo task were nonsignificant (*p* = .404, *p* = .334, respectively). Whereas the *∆R* ^2^ for ETS exposure was significant (*∆R* ^2^ = .09, *p*‐∆*R* ^2^ = .040) for the N2 component, the step 2 model was not significant (*p* = .137); hence, we could not further interpret the ETS effect in this model. ETS exposure did not explain additional variance in the P3 component (*∆R* ^2^ = .01, *p* = .621). Overall, this indicates that the extent of ETS exposure in this study did not affect brain functioning.

## 4 DISCUSSION

In the current study, we investigated the link between ETS exposure and ERP components reflecting cue reactivity, reward processing, and inhibitory control. No associations were found between the ETS exposure and ERPs of cue reactivity and inhibitory control. With respect to reward processing (i.e., both anticipation and outcome), we found a negative bivariate correlation between ETS exposure and the reward anticipation P3 showing reduced anticipatory reward sensitivity in more ETS‐exposed individuals. However, given that this result was inconsistent across bivariate and multivariate analyses, no firm conclusions can be formulated.

We did not find the expected enhanced smoking‐related cue reactivity in exposed versus nonexposed adolescents, as measured with P3 and LPP components. This indicates that attentional processing and motivational salience for smoking‐related cues were not increased in the ETS‐exposed adolescents. The incentive sensitization model suggests that substance‐related cues acquire incentive salience through the repeated use of substances (Berridge & Robinson, 2016), which in turn leads to enhanced attentional processing of motivationally salient or substance‐related cues and ultimately results in enlarged P3 and LPP amplitudes. The current results suggest that repeated exposure to ETS, in contrast to repeated active smoking, does not result in enhanced incentive salience of smoking cues. However, the LPP amplitude for smoking‐related pictures was found to be significantly larger compared with the LPP for romantic and neutral pictures in all adolescents, regardless of ETS exposure. This finding suggests that deeper attentional processing of smoking‐related pictures does occur. In combination with the negative valence ratings for the smoking cues, this may indicate that never smokers perceive these images as unpleasant. This is in line with a previous study which concluded that smokers perceive smoking cues as salient through the repeated associations with nicotine delivery, whereas never smokers perceived cigarette cues as unpleasant (Deweese, Codispoti, Robinson, Cinciripini, & Versace, 2018). The direct link between valence ratings and LPP amplitudes was, however, not observed in our study. We did, however, observed a positive association between the valence ratings for the smoking cues and ETS exposure, indicating that the higher the exposure, the more positive (i.e., less negative) smoking cues were perceived. This suggests that seeing smoking cues in their environment becomes more normal and that the cons of smoking potentially decrease. This is in line with previous work showing that children who reported a higher number of smokers in their social environment displayed more favorable smoking‐related cognitions (i.e., perceived more pros of smoking, perceived a higher safety of casual smoking, and cue‐triggered wanting to smoke; Schuck et al., 2013). In addition, it was shown that favorable smoking‐related cognitions were associated with a higher susceptibility to smoking in the future (Schuck et al., 2012). We suggest that a higher number of smokers in the social environment of the participant in combination with perceiving smoking‐related pictures as less negative while exposure increases might lead to a higher risk of smoking in the future. The current finding supports initiatives to reduce smoking in the environment of children and adolescents and thus reduce their exposure to smoking cues to reduce the risk of smoking initiation.

Regarding reward anticipation, we expected less sensitivity to monetary rewards in exposed versus nonexposed adolescents reflected in a reduced P3 amplitude for exposed adolescents (Luijten, Schellekens, Kühn, Machielse, & Sescousse, 2017). Although no difference in the anticipatory P3 component was observed between exposed and nonexposed individuals, we found that higher exposure to ETS was associated with reduced P3 amplitudes in the exposed group, suggesting reduced anticipatory reward sensitivity for nondrug (monetary) rewards. However, this association was not corrected for multiple comparisons and it was no longer significant after controlling for gender, pubertal development, familial risk, and smoking during pregnancy, which may be because of reduced power. The observed correlation is preliminary, and future research regarding ETS exposure and brain functioning in larger samples should test whether these results can be replicated. They could further focus on reward processing to determine the relative contribution of ETS exposure and other relevant factors to possible brain changes related to reward processing.

Additionally, we did not find the expected decrease in the negativity of the FRN component in exposed versus nonexposed adolescents, suggesting no deficits in reinforcement learning (i.e., negative reward prediction error) due to ETS exposure. Although no studies on the FRN in smokers have been performed, the current findings are in contrast to the findings of Parvaz et al. (2015) who reported deficits in reinforcement learning in cocaine‐addicted individuals, as indexed by the absence of FRN amplitude modulation in this group. It could be that impaired FRN modulation is a consequence of chronic drug use and that repeated exposure to ETS does not, or not yet, affect this modulation. Future studies need to explore whether impairments in FRN modulation exist before the initiation of drug use in young adults and at‐risk populations (Parvaz et al., 2015). This study addressed this issue indirectly, and its replication might suggest that FRN impairments do not yet exist before the initiation of drug use.

With respect to inhibitory control, we expected a decrease in N2 and P3 amplitudes following ETS exposure. However, no significant effects were observed, indicating no association between ETS exposure and brain activation related to inhibitory control. This is inconsistent with previous research showing reduced inhibitory control in smokers and individuals exposed to cigarette smoke prenatally (Bennett et al., 2013; Boucher et al., 2014; Holz et al., 2014; Longo et al., 2013; Luijten et al., 2014; Smith et al., 2014). In general, whether deficits related to inhibitory control should be interpreted as a consequence or cause of substance dependence is unclear. A recent study among never smokers investigated preexisting deficits related to inhibitory control and their effects of future nicotine dependence. Individuals that did develop a nicotine dependence later in life indeed show preexisting deficits related to inhibitory control before smoking initiation (Anokhin & Golosheykin, 2016). This suggests that these deficits are a cause of substance dependence rather than a consequence, which might explain why we did not find deficits in inhibitory control after ETS exposure. However, this needs to be verified in future longitudinal studies that also measure ETS exposure.

The current study has several strengths. This is the first study investigating the effects of ETS exposure on functional brain changes using ERPs while controlling for several potentially confounding factors in a relatively large sample of adolescents. Second, this study aimed to study the pure effects of ETS exposure and therefore a design with never‐smoking adolescents was necessary, ruling out possible effects of early smoking on brain functioning. Third, the ETS exposure questionnaire includes information on not only the number of smokers (Bélanger et al., 2008; Okoli et al., 2016), but also the frequency of smoking in the presence of our participants (ranging from he/she smokes but not when I am around (0) to more than five times a day (8)) to obtain a better understanding of the actual exposure in their homes and environment during the week. A recent study measuring past week exposure to smoking in the home used a similar approach to construct the ETS exposure measure and concluded that this measure is an important risk factor for adolescent smoking (Ball, Sim, & Edwards, 2018). Fourth, our study included cotinine measurements in saliva to objectively verify the ETS exposure measure. Although the self‐reported versus biologically determined ETS exposure categories were not completely overlapping, probably because saliva cotinine values capture the exposure to ETS only in the previous 1–3 days (Racicot et al., 2011), we did observe an association between self‐reported ETS exposure and biologically verified ETS exposure.

Despite these strengths, the results should be interpreted in the context of some limitations. First, within the ETS‐exposed group, the mean level of exposure was relatively low, with only few participants indicating moderate‐to‐high levels of exposure. While this distribution may be in line with the decreasing levels of ETS exposure in the Dutch adolescent population, it may also explain the absence of ETS exposure effects on brain functioning in this study. Future studies should focus on a larger and more heterogeneous sample concentrating for example on high‐risk groups and even younger adolescents (to prevent the likelihood that high‐exposed adolescents already started smoking themselves), increasing the likelihood of including participants with high levels of exposure. Future research can further improve the ETS exposure measure, by including questions on the intensity of exposure including time spent with various people in the environment and the average number of cigarettes smoked when around in addition to the frequency of exposure as included in the current study to get an even better overview of the actual exposure in the environment. Another limitation of this study is the significant difference in age between the two groups. In our analyses, we included pubertal developmental scores to account for ongoing brain maturation. Previous studies found evidence for the fact that pubertal development better describes developmental change compared with chronical age (Brumback, Arbel, Donchin, & Goldman, 2012; Herting & Sowell, 2017; Mathes, Khalaidovski, Wienke, Schmiedt‐Fehr, & Basar‐Eroglu, 2016; van Duijvenvoorde, Westhoff, de Vos, Wierenga, & Crone, 2019; Wierenga et al., 2018). Nevertheless, developmental changes may have limited our ability to observe ETS effects. To better control for confounding effects of pubertal development, future studies could include participants within a smaller age range or perform time–frequency analysis as a previous study showed that time–frequency analysis helps to define neurodevelopmental changes (Mathes et al., 2016). Moreover, some of the mothers smoked during pregnancy, resulting in 13 participants that were prenatally exposed to tobacco, although we included this information in our analyses, by including it as a covariate, it could impact our findings. The small group size of the prenatally exposed group prevented us from performing subgroup analyses. Future research investigating the effects of prenatal cigarette smoke exposure with sufficient power is, however, strongly warranted. Finally, this study was cross‐sectional, and to test whether ETS exposure over longer time periods precedes functional brain changes during adolescence, longitudinal studies that would measure ETS exposure several times during childhood and adolescence are needed.

## 5 CONCLUSION

In summary, this study is the first to investigate the association between ETS exposure and brain functioning related to smoking cue reactivity, reward processing, and inhibitory control. The findings showed no indications that ETS exposure during adolescence affects cue reactivity and inhibitory control, although it should be noted that higher ETS exposure was associated with a more positive (i.e., less negative) explicit evaluation of smoking pictures. An implication of the latter finding is that prevention efforts should focus on reducing the exposure to ETS. The results regarding reward processing, more specifically reward anticipation, are still inconclusive. Overall, our findings suggest that ETS exposure has little or no effect on brain functioning when measured with the experimental tasks selected in this study. However, since our sample included few participants with relatively high levels of exposure to ETS, this conclusion should be considered as preliminary. Future studies should focus on subgroups of adolescents with high levels of exposure to establish its effects on reward processing, as such effects could be limited to these high‐risk groups. Based on the data of this study, we can conclude that low‐to‐moderate exposure to ETS during adolescence does not result in functional brain changes related to smoking cue reactivity, reward processing, and inhibitory control.

## CONFLICT OF INTEREST

The authors declare they have no conflict of interest.

## AUTHORS’ CONTRIBUTION

ML wrote the grant for this project, designed the study, contributed to the data analysis, and edited the manuscript. JD designed the study, performed the electroencephalography recordings and data analysis, and wrote the manuscript. HS assisted in setting up the EEG preprocessing pipeline and editing the manuscript. VH assisted in participant recruitment and editing the supplementary materials. MK and RO wrote the grant for this project, designed the study, and edited the manuscript.

## Supporting information

Table S1‐S40

Fig S1

## Data Availability

Data are available via data archiving and network services of DANS: https://doi.org/10.17026/dans‐zgc‐5nbz.
