# Supplementary material for: Effects of environmental tobacco smoke exposure on brain functioning in never‐smoking adolescents
Source: Brain Behav. 2020 Jul 1;10(8):e01619. doi: 10.1002/brb3.1619 (PMC7428475; doi:10.1002/brb3.1619)
Supplement: Supplementary file 1 — Table S1‐S40 [file BRB3-10-e01619-s001.docx]

**Methods**

*Participants and procedures.* Participants were invited to the Behavioural Science Institute (BSI) lab at the Radboud University for the EEG test session. Participants were seated in a comfortable EEG chair in a sound-attenuated room. Electrodes were attached and task instructions were explained. Participants performed a Go/IfGo/NoGo task, followed by a monetary incentive delay (MID) task and a smoking cue reactivity task during EEG recording.

*Questionnaires – Pubertal development measure.* The Pubertal Development Scale (Petersen, Crockett, Richards, & Boxer, 1988) (PDS) is a questionnaire assesing body growth, pubic hair, skin changes, voice change and facial hair (for boys); and breast development and menarhe (for girls). Participants indicated on a 4 point scale whether a physical characteristic (1) not yet started, (2) barely started, (3) definitely started, and (4) seems complete. Point values are averaged for all items to give a PDS score, ranging from 1 to 4. Cronbach’s alphas were 0.807 and 0.683 for boys and girls respectively.

*Familial risk.* To obtain an estimate of the participants’ sensitivity for developing a nicotine dependence, a familial risk score was created. One of the parents filled out a questionnaire for his/herself, as well as for the other parent adressing. The questionnaire addressed three domains (1) their current smoking behaviour and frequency, (2) their level of nicotine dependence, for the period in which they smoked the heaviest (could either be now or in the past), assessed with the Fagerström test for nicotine dependence (FTND), which has proven to be a valuable tool to measure nicotine dependence in the past (Vink, Willemsen, Beem, & Boomsma, 2005) and (3) smoking behaviour of their own parents (i.e., the grandparents of the participants)

Current smoking behaviour and frequency were assessed using the question whether they smoke right now, yes(1) or no (0) and if they smoke, they were asked to report the number of cigarettes smoked per day. Answers were divided into three categories: (1) smokers who smoke less than 10 cigarettes per day, (2) smokers who smoke 10 or more cigarettes per day, (3) smokers who smoke 20 or more cigarettes per day. Responses are added to compute a score ranging from 0 to 4 for each parent.

To assess the level of nicotine dependence of the parents the FTND was used. People answered the questions of the FTND for the period in which they smoked the heaviest, because information about this period provides scores of ex-smokers and smokers of their maximum degree of nicotine dependence (Vink, Willemsen, & Boomsma, 2005). The FTND includes six questions, an example question is: ‘How soon after you wake up do you smoke your first cigarette?’. Responses are added to compute a score ranging from 0 (least dependent smokers) to 10 (most dependent), as previously described by Heatherton et al. (1991), for each parent.

Smoking behaviour of the grandparents was assessed with the following question: ‘What are the smoking habits of your father/father in law/mother/mother in law’. Answer categories were: non-smoker (1), ex-smoker (2), smoke occasionally (3), smokes 1-10 cigarettes a day (4), smokes 10 or more cigarettes a day (5). If the grandparents smoked more than 10 cigarettes per day, a score of 1 was assigned, when they smoked less, were non-smokers or ex-smokers, a score of 0 was assigned. Responses are added to compute a score ranging from 0 to 2 for each parent.

For both parents scores from the three domains were summed, resulting in two total scores (father and mother), which were subsequently summed and averaged, resulting in one score representing familial risk on a scale from 0 to 16. A higher score indicates a higher risk for developing a nicotine dependence.

*Electrophysiological recording and offline data processing.* EEG signals were recorded with a 32-channel EEG amplifier using a 32-channel actiCap system (MedCat B.V. The Netherlands). Electrodes were located on positions according to the international 10 to 20 system. For linked-reference recording a reference electrode was placed on the left mastoid bone and another electrode was placed on the right mastoid bone (electrode 22). A ground electrode was located at the AFz electrode position. Eye movements were recorded using two active electrodes (VEOG and HEOG); one below the left eye and one on the outer canthus of the left eye. Electrode impedance was kept below 20 kΩ. The signal was digitized online at 1000Hz and online filter between 0.016 and 250 Hz. Recorded EEG data was analysed using Brain Vision Analyzer. During offline data processing, the signal of every EEG electrode was re-referenced to linked mastoids. Re-referencing was followed by filtering EEG and EOG activity with a band pass of 0.10-30 Hz. After filtering, EEG data was screened by means of Raw Data Inspection. Epochs including motor and ocular artefacts, such as high frequency muscle activity, were excluded using manual inspection. If bad channels existed they were subsequently removed by the individual channel mode of the raw data inspection interface. These channels were replaced by new values per channel based on topographic interpolation, only if there were no more than four bad channels per participant. Following raw data inspection, ocular correction was applied using the signal recorded from VEOG and HEOG channels. The mean 100- ms pre-event period served as baseline. After baseline correction, average ERP waves were calculated for artefact-free trials at each scalp site for the different task conditions separately.

*EEG segmentation per task per stimulus type.* EEG data for the smoking cue reactivity task were segmented in epochs of 1400 ms (400 ms before and 1000 ms after picture onset). Based on previous literature and visual inspection of the data, P3 and LPP components were defined as the mean activity within the 250-400 and 450-1000 ms time interval and studied at parietal electrodes (P3, Pz, P4) (Piasecki, Fleming, Trela, & Bartholow, 2017). The mean number of analysable segments were 30 (range 22-32), 31 (range 24-32), 31 (range 24-32) for smoking, neutral and romantic pictures respectively. Participants with less than 20 trials for each condition were excluded from analyses. Therefore, three participants were excluded for analysis of the cue reactivity task because of too many artefacts.

EEG data for the anticipatory phase of the MID task was segmented in epochs of 1900 ms (400 ms before and 1500 ms after picture onset). Based on previous literature and visual inspection of the data, the anticipatory P3 was defined as the mean activity within the 275-500 ms time interval and studied at Pz, CP1, CP2, P3 and P4 (Broyd et al., 2012; Glazer, Kelley, Pornpattananangkul, Mittal, & Nusslock, 2018; Goldstein et al., 2006; Johnson & Donchin, 1980; Pfabigan et al., 2014; Pornpattananangkul & Nusslock, 2015). EEG data for the outcome phase of the MID task was segmented in epochs of 2000 ms (400ms before and 1600 ms after feedback was presented). Based on previous literature and visual inspection of the data, the FRN was defined as the mean activity within 200-300 ms interval and studied at the FCz, Fz, FC1 and FC2 electrodes, of which FCz and Fz are in line with previous literature (Glazer et al., 2018). The mean number of analyzable epochs for the anticipatory phase were 57 (range 46-60) for reward and 58 (range 45-60) for non-reward. The mean number of analyzable epochs for the outcome phase were 40 (range 30-49) for reward correct, 16 (range 10-22) for reward incorrect. A minimum of 20 trials for reward or non-reward during the anticipatory phase was needed for analysis and a minimum of 20 trials for correct and less than 10 for incorrect during the outcome phase were needed (Marco-Pallares, Cucurell, Münte, Strien, & Rodriguez-Fornells, 2011). Therefore two participants were excluded for all analyses, because of too many artefacts in the data. Additionaly, another five participants were excluded for analyses of the outcome phase because less than 10 segments remained.

EEG data for the Go/IfGo/NoGo task was segmented in epochs of 1200 ms (400 ms before and 800 ms after picture onset). Segments with incorrect responses (miss for Go trials or false alarm for NoGo trials were excluded for analyses. Based on previous literature and visual inspection of the data, N2 and P3 The N2 and P3 components were defined as the mean activity within the 200-320 and 320-500 ms time interval and studied at F3, F4, Fz, and FCz for the N2 and FC1, FCz, Cz and FC2 for P3 respectively (Luijten, Littel, & Franken, 2011). The mean number of analyzable Go, IfGo and NoGo epochs were 231 (range 120-249), 62 (range 28-67) and 51 (range 29-65). Participants with less than 20 trials for each condition were excluded from analyses. Therefore, two participants were excluded for analysis of the Go/IfGo/NoGo task because of too many artefacts.

**Cue reactivity**

Table S1. *Mean amplitudes cue reactivity for the P3 and LPP components for each picture type ETS exposed and non-exposed participants*

|  | | Exposed (*n* = 49)^a^ | |  | Non-exposed (*n* = 32) | |
| --- | --- | --- | --- | --- | --- | --- |
| Picture type | | *M* | *SD* |  | *M* | *SD* |
| P3 | Smoke | 5.72 | 8.09 |  | 6.92 | 7.93 |
|  | Romantic | 7.28 | 8.72 |  | 7.85 | 8.25 |
|  | Neutral | 4.98 | 7.99 |  | 5.80 | 7.22 |
|  | Diff. smoke - neutral | 0.74 | 3.46 |  | 1.13 | 3.46 |
| LPP | Smoke | 4.33 | 5.83 |  | 4.03 | 6.38 |
|  | Romantic | 2.40 | 5.84 |  | 2.49 | 6.19 |
|  | Neutral | 1.32 | 5.68 |  | 0.42 | 5.30 |
|  | Diff. smoke - neutral | 3.00 | 3.90 |  | 3.61 | 4.50 |

*Notes.* ^a^Three participants from the ETS exposed group were excluded due to insufficient EEG recording quality. Mean scores were averaged over electrodes.

Table S2. *Results cue reactivity for the P3 and LPP components including covariates*

| Predictor | | *dfs* | *F* | *p* | *Partial η^2^* |
| --- | --- | --- | --- | --- | --- |
| P3 | Group | 1, 73 | 0.23 | .63 | .003 |
|  | Picture type | 2, 146 | 3.62 | .029 | .047 |
|  | Electrode | 2, 146 | 3.42 | .036 | .045 |
|  | Picture type × Group | 2, 146 | 0.05 | .952 | .001 |
|  | Electrode × Group | 2, 146 | 0.99 | .374 | .013 |
|  | Picture Type × Electrode | 3.10, 226.00^a^ | 0.79 | .517 | .011 |
| LPP | Group | 1, 73 | 0.19 | .663 | .003 |
|  | Picture type | 2, 146 | 5.37 | .006 | .069 |
|  | Electrode | 2, 146 | 6.02 | .003 | .076 |
|  | Picture type × Group | 2, 146 | 0.51 | .603 | .007 |
|  | Electrode × Group | 2, 146 | 1.43 | .243 | .019 |
|  | Picture type × Electrode | 3.35, 244.7^b^ | 0.94 | .436 | .013 |

*Note. N* = 79.

^a,b^ Mauchly’s test of sphericity showed a significant effect (*p* <.001 for both P3 and LPP). Greenhouse-Geisser correction was used to adjust the degrees of freedom.

Table S3. *Estimated Marginal Means of picture types for P3 and LPP components after controlling for covariates*

|  | P3 component | |  | LPP component | |  |
| --- | --- | --- | --- | --- | --- | --- |
| Trial type | EMM  (Std. Error) | 95% Confidence Interval |  | EMM  (Std. Error) | 95% Confidence Interval |  |
| Smoke | 6.31 (0.93) | [4.44, 8.17] |  | 4.28 (0.69) | [2.90, 5.66] | |
| Romantic | 7.61 (0.99) | [5.64, 9.59] |  | 2.53 (0.65) | [1.23, 3.83] | |
| Neutral | 5.42 (0.91) | [3.61, 7.22] |  | 0.95 (0.64) | [-0.32, 2.22] | |

*Note.* EMM = Estimated Marginal Mean; SE = Standard Error.

Table S4. *Pairwise comparisons between picture types for P3 and LPP components after controlling for covariates*

| Comparison | | Mean  Difference^a^ | Std.  Error | *p* | 95% Confidence Interval |
| --- | --- | --- | --- | --- | --- |
| P3 | Smoke vs Romantic | 1.31 | 0.37 | .002 | [0.39, 2.22] |
|  | Smoke vs Neutral | 0.89 | 0.41 | .096 | [-0.11, 1.89] |
|  | Romantic vs Neutral | 2.20 | 0.41 | < .001 | [1.18, 3.21] |
| LPP | Smoke vs Romantic | 1.75 | 0.43 | < .001 | [0.69, 2.81] |
|  | Smoke vs Neutral | 3.33 | 0.49 | < .001 | [2.13, 4.53] |
|  | Romantic vs Neutral | 1.58 | 0.45 | .002 | [0.48, 2.68] |

*Note.* ^a^Mean difference between estimated marginal means.

Bonferroni adjustment was used to correct for multiple comparisons.

Table S5. *Results cue reactivity for the P3 and LPP components, models excluding covariates*

| Predictor | | *dfs* | *F* | *p* | *Partial η^2^* |
| --- | --- | --- | --- | --- | --- |
| P3 | Group | 1, 79 | 0.23 | .630 | .003 |
|  | Picture type | 2, 158 | 15.99 | < .001 | .168 |
|  | Electrode | 2, 158 | 77.64 | < .001 | .496 |
|  | Picture type × Group | 2, 158 | 0.35 | .703 | .004 |
|  | Electrode × Group | 2, 158 | 1.64 | .197 | .020 |
|  | Picture Type × Electrode | 3.06, 241.47^a^ | 1.72 | .162 | .021 |
| LPP | Group | 1,79 | .09 | .764 | .001 |
|  | Picture type | 2, 158 | 27.23 | < .001 | .256 |
|  | Electrode | 2, 158 | 98.81 | < .001 | .556 |
|  | Picture type × Group | 2, 158 | 0.62 | .540 | .008 |
|  | Electrode × Group | 2, 158 | 1.73 | .181 | .021 |
|  | Picture type × Electrode | 3.36, 265.41^b^ | 2.02 | .104 | .025 |

*Notes. N* = 81. ^a,b^ Mauchly’s test of sphericity showed a significant effect (*p* <.001 for both P3 and LPP). Greenhouse-Geisser correction was used to adjust the degrees of freedom.

Table S6. *Descriptives of picture types for P3 and LPP components, models excluding covariates*

|  | P3 component | |  | LPP component | |
| --- | --- | --- | --- | --- | --- |
| Picture type | EMM  (Std. Error) | 95% Confidence Interval |  | EMM  (Std. Error) | 95% Confidence Interval |
| Smoke | 6.32 (0.91) | [4.50, 8.14] |  | 4.18 (0.69) | [2.81, 5.55] |
| Romantic | 7.56 (0.97) | [3.65, 7.13] |  | 2.45 (0.68) | [1.09, 3.80] |
| Neutral | 5.39 (0.87) | [5.63, 9.50] |  | 0.87 (0.63) | [-0.38, 2.12] |

*Note.* EMM = Estimated Marginal Mean; SE = Standard Error.

Table S7. *Pairwise comparisons between picture types for P3 and LPP, models excluding covariates*

| Comparison | | Mean  Difference^a^ | Std.  Error | *p* | 95% Confidence Interval |
| --- | --- | --- | --- | --- | --- |
| P3 | Smoke vs Romantic | 1.24 | 0.36 | .003 | [0.37, 2.12] |
|  | Smoke vs Neutral | 0.93 | 0.39 | .061 | [-0.03, 1.90] |
|  | Romantic vs Neutral | 2.18 | 0.41 | < .001 | [1.19, 3.17] |
| LPP | Smoke vs Romantic | 1.73 | 0.42 | < .001 | [0.70, 2.76] |
|  | Smoke vs Neutral | 3.31 | 0.47 | < .001 | [2.16, 4.46] |
|  | Romantic vs Neutral | 1.57 | 0.45 | .002 | [0.47, 2.68] |

*Note.* ^a^Mean difference between estimated marginal means. Bonferroni adjustment was used to correct for multiple comparisons.

Table S8*. Hierarchical regression model: P3 difference scores smoke minus neutral pictures*

| Predictor | | *B (Std. Error)* | *β* | *t* | *F* | *DFs* | *R^2^* | *∆R^2^* | *p* |
| --- | --- | --- | --- | --- | --- | --- | --- | --- | --- |
| Step 1 | |  |  |  | 1.21 | 4, 47 | .094 | .094 | .317 |
|  | Constant | 2.40 (2.58) |  | 0.93 |  |  |  |  | .358 |
|  | Gender | -0.62 (1.20) | -0.09 | -0.52 |  |  |  |  | .606 |
|  | Smoking during preg | -2.34 (1.31) | -0.29 | -1.79 |  |  |  |  | .080 |
|  | Familial risk | 0.24 (0.26) | 0.15 | 0.92 |  |  |  |  | .364 |
|  | Pubertal status | -0.45 (0.92) | -0.08 | -0.48 |  |  |  |  | .631 |
| Step 2 | |  |  |  | 0.96 | 5, 46 | .095 | .001 | .452 |
|  | Constant | 2.41 (2.61) |  | 0.93 |  |  |  |  | .360 |
|  | Gender | -0.61 (1.21) | -0.09 | -0.50 |  |  |  |  | .620 |
|  | Smoking during preg | -2.3 (1.33) | -0.28 | -1.73 |  |  |  |  | .091 |
|  | Familial risk | 0.25 (0.27) | 0.15 | 0.93 |  |  |  |  | .359 |
|  | Pubertal status | -0.42 (0.94) | -0.08 | -0.45 |  |  |  |  | .657 |
|  | ETS exposure | -0.02 (0.07) | -0.03 | -0.21 |  |  |  |  | .833 |

*Notes. N* = 52. Dependent variable is the difference score in P3 amplitudes between smoke and neutral pictures, averaged over electrodes.

Table S9*. Hierarchical regression model: LPP difference scores smoke minus neutral pictures*

| Predictor | | *B (Std. Error)* | *β* | *t* | *F* | *DFs* | *R^2^* | *∆R^2^* | *p* |
| --- | --- | --- | --- | --- | --- | --- | --- | --- | --- |
| Step 1 | |  |  |  | 0.43 | 4, 47 | .04 | .04 | .790 |
|  | Constant | 6.00 (2.00) |  | 2.00 |  |  |  |  | .051 |
|  | Gender | 0.72 (1.39) | 0.09 | 0.52 |  |  |  |  | .606 |
|  | Smoking during preg | -0.46 (1.51) | -0.05 | -0.31 |  |  |  |  | .761 |
|  | Familial risk | 0.12 (0.30) | 0.07 | 0.38 |  |  |  |  | .702 |
|  | Pubertal status | -1.20 (1.07) | -0.20 | -1.12 |  |  |  |  | .268 |
| Step 2 | |  |  |  | 0.40 | 5, 46 | .04 | .006 | .848 |
|  | Constant | 5.96 (3.01) |  | 1.98 |  |  |  |  | .054 |
|  | Gender | 0.67 (1.41) | 0.09 | 0.48 |  |  |  |  | .636 |
|  | Smoking during preg | -0.58 (1.54) | -0.06 | -0.37 |  |  |  |  | .711 |
|  | Familial risk | 0.10 (0.31) | 0.05 | 0.31 |  |  |  |  | .759 |
|  | Pubertal status | -1.28 (1.09) | -0.21 | -1.18 |  |  |  |  | .246 |
|  | ETS exposure | 0.05 (0.08) | 0.09 | 0.56 |  |  |  |  | .580 |

*Notes. N* = 52. Dependent variable is the difference score in LPP amplitudes between smoke and neutral pictures, averaged over electrodes.

Table S10*. Separate regression models: Difference scores smoke-neutral for P3 and LPP components, excluding covariates*

| Predictor | | *B (Std. Error)* | *β* | *t* | *dfs* | *F* | *R^2^* | *p* |
| --- | --- | --- | --- | --- | --- | --- | --- | --- |
| P3 diff. smoke – neutral^a^ | |  |  |  | 1, 50 | 0.50 | .01 | .481 |
|  | Constant | 1.14 (0.73) |  | 1.55 |  |  |  | .127 |
|  | ETS exposure | -0.05 (0.07) | -1.00 | -0.71 |  |  |  | .481 |
| LPP diff. smoke – neutral^b^ | |  |  |  | 1, 50 | 0.14 | .003 | .706 |
|  | Constant | 2.76 (0.82) |  | 3.34 |  |  |  | .002 |
|  | ETS exposure | 0.03 (0.08) | 0.05 | 0.38 |  |  |  | .706 |

*Notes.* N = 52. In each model, the only predictor was the continuous measure of ETS exposure.

^a.^Dependent variable is the difference score in P3 amplitude between smoke and neutral pictures. ^b.^Dependent variable is the difference score in LPP amplitude between smoke and neutral pictures. Difference scores were averaged over electrodes.

Table S11. *Valence and Arousal Ratings for the neutral, smoke and romantic pictures of thecue-reactivity task for Non-Exposed and Exposed group.*

|  | Non-Exposed (N=32) | | | Exposed (N=52) | | |
| --- | --- | --- | --- | --- | --- | --- |
| Ratings valence/arousal | Mean | SD | Range | Mean | SD | Range |
| Valence Neutral | 44,65 | 24,36 | 0 – 92 | 40,31 | 21,75 | 0 – 98,75 |
| Valence Smoke | -65,43 | 23,28 | -100 – 31,69 | -58,24 | 31,10 | -100 – 7,44 |
| Valence Romantic | 71,78 | 23,54 | 0 – 100 | 66,11 | 25,44 | 0 – 100 |
| Arousal Neutral | 35,52 | 20,67 | 3,13 – 93 | 30,14 | 21,97 | 0 – 89,63 |
| Arousal Smoke | 38,31 | 24,28 | 1,25 – 93,38 | 39,97 | 26,04 | 0,69 – 94,06 |
| Arousal Romantic | 53,09 | 28,66 | 0 – 100 | 47,58 | 28,00 | 1,25 – 100 |

Table S12. *Results valence and arousal ratings of pictures of cue reactivity task*

| Predictor | | *dfs* | *F* | *p* | *Partial η^2^* |
| --- | --- | --- | --- | --- | --- |
| Valence | Picture type | 1, 277 | 604,997 | .000 | .881 |
|  | Picture type × Group | 1, 277 | 1,584 | .214 | .019 |
| Arousal | Picture type | 1,650 | 10,124 | .000 | .110 |
|  | Picture type × Group | 1,650 | .543 | .549 | .007 |

Table S13. *Estimated Margianal Means of Picture Type for valence and arousal ratings of cue reactivity task*

|  | Valence | |  | Arousal | |  |
| --- | --- | --- | --- | --- | --- | --- |
| Picture Type | EMM  (Std. Error) | 95% Confidence Interval |  | EMM  (Std. Error) | 95% Confidence Interval |  |
| Neutral | 42.48 (2.56) | [37.39, 47.57] |  | 32,83 (2.41) | [28.03, 37.63] | |
| Smoke | -61.84 (3.55) | [-68.88, -54.79] |  | 39,14 (2.85) | [33.47 44.82] | |
| Romantic | 68.94 (2.78) | [63.41, 74.47] |  | 50.34 (3.17) | [44.02, 56.65] | |

*Note.* EMM = Estimated Marginal Mean; SE = Standard Error.

Table S14*. Pairwise comparisons between picture types for valence and arousal ratings*

*of cue reactivity task*

| Comparison | | Mean  Difference^a^ | Std.  Error | *p* | 95% Confidence Interval |
| --- | --- | --- | --- | --- | --- |
| Valence | Neutral vs Smoke | 104.315 | 4.60 | .000 | [-93.08, 115.56] |
|  | Neutral vs Romantic | -26.47 | 1.98 | .000 | [-31.31, -21,62] |
|  | Smoke vs Romantic | -130.78 | 4.73 | .000 | [-142.33, -119, 23] |
| Arousal | Neutral vs Smoke | -6.31 | 3.37 | .194 | [-14.54, 1.93] |
|  | Neutral vs Romantic | -17.51 | 3.55 | .000 | [-26.17, -8.84] |
|  | Smoke vs Romantic | -11.19 | 4.76 | .063 | [-0.44, 22.83] |

*Note.* ^a^Mean difference between estimated marginal means.

Bonferroni adjustment was used to correct for multiple comparisons.

**Monetary Incentive Delay task**

Table S15. *Mean amplitudes MID for the P3 and FRN components for each trial type for ETS exposed and non-exposed participants*

|  | | Exposed | | |  | Non-exposed | | |
| --- | --- | --- | --- | --- | --- | --- | --- | --- |
| Trial type | | *n* | *M* | *SD* |  | *n* | *M* | *SD* |
| Behavioral reaction times | |  |  |  |  |  |  |  |
|  | Reward | 52 | 181.46 | 18.64 |  | 32 | 188.97 | 25.71 |
|  | Non-reward | 52 | 189.10 | 20.40 |  | 32 | 197.19 | 26.82 |
| P3 | Reward | 50 | 4.40 | 4.32 |  | 32 | 4.96 | 3.80 |
|  | Non-reward | 50 | 1.70 | 3.53 |  | 32 | 2.42 | 4.46 |
|  | Diff. Reward – Non-reward | 50 | 2.70 | 3.11 |  | 32 | 2.54 | 3.13 |
| FRN | Reward incorrect | 47 | 12.57 | 6.83 |  | 30 | 11.38 | 7.63 |
|  | Reward correct | 50 | 14.22 | 5.57 |  | 32 | 13.47 | 7.46 |
|  | Diff. Reward_incorrect_ – Reward_correct_ | 47 | -1.68 | 5.88 |  | 30 | -1.65 | 5.62 |

*Notes.* Mean scores for ERP outcome measures were averaged over electrodes. Behavioral reaction times are reported in miliseconds.

Table S16. *Monetary Incentive Delay task:* *Reaction times results, including and excluding covariates*

|  | Model controlled for covariates^a^ | | | |  | Model excluding covariates^b^ | | | |
| --- | --- | --- | --- | --- | --- | --- | --- | --- | --- |
| Predictor | *dfs* | *F* | *p* | *Partial η^2^* |  | *dfs* | *F* | *p* | *Partial η^2^* |
| Group | 1, 76 | 2.99 | .088 | .038 |  | 1, 82 | 2.57 | .113 | .030 |
| Trial type | 1, 76 | 1.14 | .289 | .015 |  | 1, 82 | 44.23 | <.001 | .350 |
| Group × Trial type | 1, 76 | 0.14 | .707 | .002 |  | 1, 82 | 0.06 | .811 | .001 |

*Note.* ^a^*N* = 82; ^b^*N* = 84.

Table S17. *Monetary Incentive Delay task:* *Follow-up RTs reward versus non-reward trials, excluding covariates*

|  | Excluding covariates | | |
| --- | --- | --- | --- |
| Trial type | EMM  (Std. Error) | 95% Confidence Interval | *p* |
| Reward | 185.22 (2.43) | [180.39, 190.04] |  |
| Non-reward | 193.14 (2.59) | [187.99, 198.29] |  |
| Mean difference |  | [5. 51, 10.35] | < .001 |

*Note.* EMM = Estimated Marginal Mean.

Table S18*. Results MID for the P3 and FRN components including covariates*

| Predictor | | *Dfs*^c^ | *F* | *p* | *Partial η^2^* |
| --- | --- | --- | --- | --- | --- |
| P3^a^ | Group | 1, 74 | 0.26 | .610 | .004 |
|  | Reward | 1, 74 | 9.68 | .003 | .116 |
|  | Electrode | 2.52, 186.68 | 2.84 | .049 | .037 |
|  | Reward × Group | 1, 74 | 0.46 | .502 | .006 |
|  | Electrode × Group | 2.52, 186.68 | 2.16 | .105 | .028 |
|  | Reward × Electrode | 3.00, 221.72 | 0.72 | .544 | .010 |
| FRN^b^ | Group | 1,69 | 3.502 | .066 | .048 |
|  | Reward_Outcome | 1, 69 | 1.779 | .187 | .025 |
|  | Electrode | 1.87, 128.69 | 2.745 | .019 | .038 |
|  | Reward_Outcome x Group | 1,69 | .407 | .525 | .006 |
|  | Electrode x Group | 1.87, 128.69 | 1.988 | .080 | .028 |
|  | Reward_Feedback × Electrode | 2.22, 153.19 | .700 | .624 | .010 |

*Notes:* ^a^ *N* = 80; ^b,^ *N* = 73; ^c^ Mauchly’s test of sphericity showed significant effect for electrode in both models. Greenhouse-Geisser correction was used to adjust the degrees of freedom.

Table S19. MID: *Estimated Marginal Means and pairwise comparisons after controlling for covariates for P3 and FRN*

| Trial Type | | EMM  (Std. Error) | Mean Difference  (Std. Error) | *p* | 95% Confidence Interval |
| --- | --- | --- | --- | --- | --- |
| P3 | Reward | 4.66 (0.46) |  |  | [3.75, 5.57] |
|  | Non-reward | 2.01 (0.45) |  |  | [1.11, 2.91] |
|  | Reward versus non-reward |  | 2.65 (0.36) | < .001 | [1.93, 3.37] |
| FRN | Reward incorrect | 11.65 (0.81) |  |  | [10.04, 13.26] |
|  | Reward correct | 13.28 (0.72) |  |  | [11.84, 14.72] |
|  | Reward incorrect versus Reward ddcorrect |  | -1.63 (0.65) | .014 | [-2.92, -.34] |
|  | Group ETS exposed | 13.88 (0.89) |  |  | [12.10, 15.65] |
|  | Group ETS non-exposed | 11.05 (1.15) |  |  | [8.76, 13.33] |
|  | ETS exposed versus non- ddexposed |  | 2.83 (1.51) | .066 | [-.187, 5.84] |

*Note.* EMM = Estimated Marginal Mean. Bonferroni adjustment was used to correct for multiple comparisons.

Table S20*. Results MID for the P3 and FRN components, models excluding covariates*

| Predictor | | *Dfs*^c^ | *F* | *p* | *Partial η^2^* |
| --- | --- | --- | --- | --- | --- |
| P3^a^ | Group | 1, 80 | 0.58 | .449 | .007 |
|  | Reward | 1, 80 | 55.06 | < .001 | .408 |
|  | Electrode | 2.54, 203.54 | 11.92 | < .001 | .130 |
|  | Reward × Group | 1, 80 | 0.52 | .820 | .001 |
|  | Electrode × Group | 2.52, 186.68 | 2.38 | .081 | .029 |
|  | Reward × Electrode | 3.01, 241.13 | 4.81 | .003 | .057 |
| FRN^b^ | Group | 1, 75 | 0.714 | .401 | .009 |
|  | Reward_Outcome | 1, 75 | 6.087 | .016 | .075 |
|  | Electrode | 1.89, 141.78 | 58.99 | <.001 | .440 |
|  | Reward_Outcome x Group | 1, 75 | 0.001 | .979 | .000 |
|  | Electrode x Group | 1.89, 141.78 | 0.969 | .378 | .013 |
|  | Reward_Feedback × Electrode | 2.19, 164.10 | 1.421 | .216 | .019 |

*Notes:* ^a^ *N* = 82; ^b^ *N* = 75; ^c^ Mauchly’s test of sphericity showed significant effects for electrode in both models. Greenhouse-Geisser correction was used to adjust the degrees of freedom.

Table S21. MID: *Estimated Marginal Means and pairwise comparisons, excluding covariates for P3 and FRN*

| Trial Type | | EMM  (Std. Error) | Mean Difference  (Std. Error) | *p* | 95% Confidence Interval |
| --- | --- | --- | --- | --- | --- |
| P3 | Reward | 4.68 (0.47) |  |  | [3.75, 5.61] |
|  | Non-reward | 2.06 (0.33) |  |  | [1.18, 2.95] |
|  | Reward versus non-reward |  | 2.62 (0.35) | < .001 | [1.92, 3.32] |
| FRN | Reward incorrect | 11.98 (0.84) |  |  | [10.31, 13.64] |
|  | Reward correct | 13.64 (0.75) |  |  | [12.15, 15.14] |
|  | Reward incorrect versus fffeReward correct |  | -1.67 (0.68) | .016 | [-3.011, -0.32] |
|  | Group ETS exposed | 13.42 (0.89) |  |  | [10.02, 12.78] |
|  | Group ETS non-exposed | 12.20 (1.12) |  |  | [9.45, 12.41] |
|  | ETS exposed versus non- eeeexposed |  | 1.22 (1.44) | .401 | [-1.65, 4.08] |

*Note.* EMM = Estimated Marginal Mean. Bonferroni adjustment was used to correct for multiple comparisons.

Table S22*. MID: hierarchical regression model P3 Reward minus non-reward*

| Predictor | | *B (Std. Error)* | *β* | *t* | *dfs* | *F* | *R^2^* | *∆R^2^* | *p* |
| --- | --- | --- | --- | --- | --- | --- | --- | --- | --- |
| Step 1 | |  |  |  | 4, 44 | 1.60 | .13 | .13 | .192 |
|  | Constant | 7.51 (2.49) |  | 3.02 |  |  |  |  | .004 |
|  | Gender | -0.05 (1.10) | -.01 | -0.04 |  |  |  |  | .967 |
|  | Smoking during preg | -0.86 (1.22) | -.11 | -0.71 |  |  |  |  | .485 |
|  | Familial risk | -0.18 (0.24) | -.13 | -0.77 |  |  |  |  | .443 |
|  | Pubertal status | -1.33 (0.89) | -.26 | -1.50 |  |  |  |  | .141 |
| Step 2 | |  |  |  | 5, 43 | 1.74 | .17 | .04 | .147 |
|  | Constant | 7.54 (2.46) |  | 3.07 |  |  |  |  | .004 |
|  | Gender | 0.08 (1.09) | .01 | 0.08 |  |  |  |  | .940 |
|  | Smoking during preg | -0.62 (1.22) | -.08 | -0.51 |  |  |  |  | .616 |
|  | Familial risk | -0.14 (0.24) | -.10 | -0.61 |  |  |  |  | .547 |
|  | Pubertal status | -1.16 (0.88) | -.22 | -1.31 |  |  |  |  | .196 |
|  | ETS exposure | -0.09 (0.63) | -.22 | -1.46 |  |  |  |  | .152 |

*Notes. N* = 49. Dependent variable is the difference score in P3 amplitude between Reward and Non-reward trials, averaged over electrodes.

Table S23*. MID: hierarchical regression model FRN Reward incorrect minus reward correct*

| Predictor | | *B (Std. Error)* | *β* | *t* | *dfs* | *F* | *R^2^* | *∆R^2^* | *p* |
| --- | --- | --- | --- | --- | --- | --- | --- | --- | --- |
| Step 1 | |  |  |  | 4, 41 | 4.37 | .30 | .30 | .005 |
|  | Constant | 1.43 (4.21) |  | 0.34 |  |  |  |  | .735 |
|  | Gender | -6.40 (1.95) | -.53 | -3.28 |  |  |  |  | .002 |
|  | Smoking during preg | 2.77 (2.14) | .19 | 1.30 |  |  |  |  | .202 |
|  | Familial risk | -0.31 (0.40) | -.12 | -0.77 |  |  |  |  | .448 |
|  | Pubertal status | 0.35 (1.51) | .04 | 0.23 |  |  |  |  | .817 |
| Step 2 | |  |  |  | 5, 40 | 3.89 | .33 | .03 | .006 |
|  | Constant | 1.37 (4.18) |  | 0.33 |  |  |  |  | .744 |
|  | Gender | -6.66 (1.95) | -.55 | -3.42 |  |  |  |  | .001 |
|  | Smoking during preg | 2.37 (2.14) | .16 | 1.11 |  |  |  |  | .275 |
|  | Familial risk | -0.37 (0.40) | -.14 | -0.93 |  |  |  |  | .360 |
|  | Pubertal status | 0.12 (1.51) | .01 | 0.08 |  |  |  |  | .939 |
|  | ETS exposure | 0.14 (0.11) | .18 | 1.29 |  |  |  |  | .203 |

*Notes. N* = 46. Dependent variable is the difference score in FRN amplitude between incorrect and correct reward trials, averaged over electrodes.

Table S24*. MID task: Separate regression models for P3 and FRN, excluding covariates*

| Predictor | | *B (Std. Error)* | *β* | *t* | *dfs* | *F* | *R^2^* | *P* |
| --- | --- | --- | --- | --- | --- | --- | --- | --- |
| P3 diff. R – NR^a^ | |  |  |  | 1, 48 | 4.39 | .08 | .042 |
|  | Constant | 3.75 (0.66) |  | 5.70 |  |  |  | < .001 |
|  | ETS exposure | -0.12 (0.06) | -.29 | -2.09 |  |  |  | .042 |
| FRN diff. R_incorrect_ – R_correct_ ^b^ | |  |  |  | 1, 45 | 0.06 | .00 | .803 |
|  | Constant | -1.93 (1.32) |  | -1.47 |  |  |  | .149 |
|  | ETS exposure | 0.03 (0.12) | 0.04 | 0.25 |  |  |  | .803 |

*Notes.* ^a^ N = 50, ^b^ N=47. In each model, the only predictor was the continuous measure of ETS exposure.

Difference scores were averaged over electrodes.

**Go/Nogo**

Table S25. *Mean amplitudes inhibitory control for the N2 and P3 components for each trial type for ETS exposed and non-exposed participants*

|  | | Exposed (*n* = 51)^a^ | |  | Non-exposed (*n* = 32) | |
| --- | --- | --- | --- | --- | --- | --- |
| Trial type | | *M* | *SD* |  | *M* | *SD* |
| Behavioral accuracy | |  |  |  |  |  |
|  | Go | 99.01 | 4.60 |  | 99.18 | 1.18 |
|  | IfGo | 98.74 | 4.82 |  | 99.35 | 1.20 |
|  | NoGo | 81.49 | 10.49 |  | 79.71 | 7.95 |
| N2 | Go | 0.93 | 3.18 |  | 0.86 | 3.49 |
|  | IfGo | -2.55 | 4.06 |  | -2.44 | 3.31 |
|  | NoGo | -2.33 | 4.68 |  | -2.82 | 4.71 |
|  | Diff. NoGo - Go | -3.27 | 3.58 |  | -3.68 | 4.00 |
| P3 | Go | 3.35 | 3.70 |  | 4.24 | 4.79 |
|  | IfGo | 8.08 | 4.26 |  | 8.06 | 7.12 |
|  | NoGo | 14.02 | 6.51 |  | 14.91 | 7.55 |
|  | Diff. NoGo - Go | 10.66 | 5.88 |  | 10.66 | 5.94 |

*Notes.* ^a^ One participant from the ETS exposed group was excluded due to insufficient EEG recording quality; *n* = 52 for the behavioral accuracy scores in the Exposed group. Mean scores were averaged over electrodes.

Table S26. *Go/IfGo/NoGo task:* *Behavioral* *accuracy results, including and excluding covariates*

|  | Model controlled for covariates^a^ | | | |  | Model excluding covariates^b^ | | | |
| --- | --- | --- | --- | --- | --- | --- | --- | --- | --- |
| Predictor | *dfs* | *F* | *p* | *Partial η^2^* |  | *dfs* | *F* | *p* | *Partial η^2^* |
| Group | 1, 75 | 3.68 | .059 | .047 |  | 1, 81 | 0.86 | .357 | .010 |
| Trial type | 1.02, 76.43 | 9.64 | .003 | .114 |  | 1.02, 82.39 | 302.14 | < .001 | .789 |
| Group × Trial type | 1.02, 76.43 | 2.89 | .093 | .037 |  | 1.02, 82.39 | 0.41 | .528 | .005 |

*Note.* ^a^*N* = 81; ^b^*N* = 83; Mauchly’s test of sphericity showed a significant effect of trial type for both models (both *ps* <.001). Greenhouse-Geisser correction was used to adjust the degrees of freedom.

Table S27. *Go/IfGo/NoGo task:* *Estimated marginal means of accuracy on trial types, with and without covariates*

|  | Controlled for covariates | |  | Excluding covariates | |
| --- | --- | --- | --- | --- | --- |
| Trial type | EMM  (Std. Error) | 95% Confidence Interval |  | EMM  (Std. Error) | 95% Confidence Interval |
| Go | 99.43 (0.10) | [99.24, 99.62] |  | 99.42 (0.09) | [99.23, 99.60] |
| IfGo | 99.38 (0.14) | [99.10, 99.65] |  | 99.37 (0.14) | [99.10, 99.64] |
| NoGo | 80.14 (1.07) | [78.01, 82.27] |  | 80.51 (1.09) | [78.34, 82.67] |

*Note.* EMM = Estimated Marginal Mean

Table S28. *Go/IfGo/NoGo task:* *Pairwise comparisons of accuracy between trial types, with and without covariates*

|  | Controlled for covariates | | |  | Excluding covariates | | |
| --- | --- | --- | --- | --- | --- | --- | --- |
| Comparison | Mean  Difference (Std. Error)^a^ | *p* | 95% CI |  | Mean  Difference (Std. Error)^b^ | *p* | 95% CI |
| Go vs IfGo | 0.06 (0.12) | 1.000 | [-0.24, 0.36] |  | 0.05 (0.12) | 1.000 | [-0.24, 0.34] |
| Go vs NoGo | 19.29 (1.05) | < .001 | [16.71, 21.88] |  | 18.91 (1.07) | < .001 | [16.29, 21.53] |
| IfGo vs NoGo | 19.24 (1.08) | < .001 | [16.60, 21.88] |  | 18.86 (1.09) | < .001 | [16.19. 21.54] |

*Note.* ^a^Mean difference between estimated marginal means. Bonferroni adjustment was used to correct for multiple comparisons.

Table S29*. Go/IfGo/NoGo task: Correlations between ETS exposure and accuracy on each of the trial types (n = 52)*

|  | 1. | 2. | 3. |
| --- | --- | --- | --- |
| 1. ETS exposure (continuous) |  |  |  |
| 2. Go accuracy | .13 |  |  |
| 3. IfGo accuracy | .15 | .97** |  |
| 4. NoGo accuracy | .09 | -.04 | -.10 |

*Notes. ** *p < .05* ** *p* < .01 *** *p* < .001 (two-tailed)

Table S30*. Results inhibitory control for the N2 and P3 components including covariates*

| Predictor | | *dfs*^a^ | *F* | *p* | *Partial η^2^* |
| --- | --- | --- | --- | --- | --- |
| N2 | Group | 1, 75 | 0.53 | .468 | .007 |
|  | Trial type | 1.75, 131.27 | 1.76 | .180 | .023 |
|  | Electrode | 2.40, 180.19 | 5.22 | .004 | .065 |
|  | Trial type × Group | 1.75, 131.27 | 1.75 | .183 | .019 |
|  | Electrode × Group | 2.40, 180.19 | 0.56 | .605 | .007 |
|  | Trial Type × Electrode | 3.63, 272.09 | 0.57 | .617 | .007 |
| P3 | Group | 1, 75 | 0.09 | .764 | .001 |
|  | Trial type | 1.58, 118.45 | 21.94 | < .001 | .226 |
|  | Electrode | 2.18, 163.57 | 7.98 | < .001 | .096 |
|  | Trial type × Group | 1.58, 118.45 | 0.69 | .471 | .009 |
|  | Electrode × Group | 2.18, 163.57 | 0.40 | .693 | .005 |
|  | Trial type × Electrode | 4.04, 303.13 | 3.23 | .013 | .041 |

*Notes: N* = 81; ^a^ Mauchly’s test of sphericity showed significant effect for all within-subject factors of both N2 and P3 components. Greenhouse-Geisser correction was used to adjust the degrees of freedom.

Table S31*. Results inhibitory control for the N2 and P3 components, models excluding covariates*

| Predictor | | *dfs*^a^ | *F* | *p* | *Partial η^2^* |
| --- | --- | --- | --- | --- | --- |
| N2 | Group | 1, 81 | 0.04 | .851 | .000 |
|  | Trial type | 1.71, 138.75 | 61.39 | < .001 | .431 |
|  | Electrode | 2.39, 193.31 | 26.79 | < .001 | .249 |
|  | Trial type × Group | 1.71, 138.75 | 0.35 | .669 | .004 |
|  | Electrode × Group | 2.39, 193.31 | 0.81 | .466 | .010 |
|  | Trial Type × Electrode | 3.70, 299.76 | 5.03 | .001 | .058 |
| P3 | Group | 1, 81 | 0.28 | .596 | .003 |
|  | Trial type | 1.58, 128.31 | 192.34 | < .001 | .704 |
|  | Electrode | 2.22, 180.00 | 59.18 | < .001 | .422 |
|  | Trial type × Group | 1.58, 128.31 | 0.46 | .587 | .006 |
|  | Electrode × Group | 2.22, 180.00 | 0.32 | .751 | .004 |
|  | Trial type × Electrode | 3.83, 310.56 | 16.65 | < .001 | .171 |

*Notes. N* = 83; ^a^ Mauchly’s test of sphericity showed significant effect for all within-subject factors of both N2 and P3 components. Greenhouse-Geisser correction was used to adjust the degrees of freedom.

Table S32. GoNoGo: *Estimated Marginal Means of picture types for the P3 component, after controlling for covariates*

|  | P3 component | |
| --- | --- | --- |
| Trial type | EMM  (Std. Error) | 95% Confidence Interval |
| Go | 3.77 (0.47) | [2.84, 4.70] |
| IfGo | 8.03 (0.63) | [6.78, 9.28] |
| NoGo | 14.26 (0.77) | [12.73, 15.79] |

*Note.* EMM = Estimated Marginal Mean.

Table S33. GoNoGo: *Estimated Marginal Means of picture types for N2 and P3 components excluding covariates*

|  | N2 component | |  | P3 component | |
| --- | --- | --- | --- | --- | --- |
| Trial type | EMM  (Std. Error) | 95% Confidence Interval |  | EMM  (Std. Error) | 95% Confidence Interval |
| Go | 0.90 (0.37) | [0.16, 1.64] |  | 3.80 (0.47) | [2.87, 4.74] |
| IfGo | -2.50 (0.43) | [-3.35, -1.65] |  | 8.07 (0.68) | [6.83, 9.31] |
| NoGo | -2.57 (0.53) | [-3.63, -1.52] |  | 14.47 (0.78) | [12.91, 16.02] |

*Note.* EMM = Estimated Marginal Mean; SE = Standard Error;

Table S34. GoNoGo: *Pairwise comparisons between trial types for N2 and P3 components in- and excluding covariates*

| Comparison | | Mean  Difference^a^ | Std.  Error | *p* | 95% Confidence Interval |
| --- | --- | --- | --- | --- | --- |
| N2 excl. covariates | Go vs IfGo | 3.39 | 0.30 | < .001 | [2.66, 4.13] |
|  | Go vs NoGo | 3.47 | 0.42 | < .001 | [2.44, 4.51] |
|  | IfGo vs NoGo | 0.08 | 0.34 | 1.00 | [-0.91, 0.75] |
| P3 excl. covariates | Go vs IfGo | 4.26 | 0.43 | < .001 | [3.22, 5.31] |
|  | Go vs NoGo | 10.66 | 0.67 | < .001 | [9.04, 12.29] |
|  | IfGo vs NoGo | 6.40 | 0.52 | < .001 | [5.12, 7.68] |
| P3 incl. covariates | Go vs IfGo | 4.27 | 0.41 | < .001 | [3.24, 5.30] |
|  | Go vs NoGo | 10.49 | 0.66 | < .001 | [8.87, 12.11] |
|  | IfGo vs NoGo | 6.23 | 0.52 | < .001 | [4.95, 7.50] |

*Note.* ^a^Mean difference between estimated marginal means.

Bonferroni adjustment was used to correct for multiple comparisons.

Table S35*. Go/Nogo: hierarchical regression model N2 Nogo minus Go*

| Predictor | | *B (Std. Error)* | *Β* | *t* | *dfs* | *F* | *R^2^* | *∆R^2^* | *p* |
| --- | --- | --- | --- | --- | --- | --- | --- | --- | --- |
| Step 1 | |  |  |  | 4, 45 | 1.03 | .08 | .08 | .404 |
|  | Constant | 1.94 (2.83) |  | 0.69 |  |  |  |  | .497 |
|  | Gender | 0.35 (1.32) | .05 | 0.27 |  |  |  |  | .791 |
|  | Smoking during preg | 0.31 (1.47) | .04 | 0.21 |  |  |  |  | .833 |
|  | Familial risk | -0.25 (0.28) | -.15 | -0.90 |  |  |  |  | .374 |
|  | Pubertal status | -1.61 (1.02) | -.28 | -1.59 |  |  |  |  | .119 |
| Step 2 | |  |  |  | 5, 44 | 1.78 | .17 | .08 | .137 |
|  | Constant | 1.79 (2.72) |  | .66 |  |  |  |  | .514 |
|  | Gender | 0.14 (1.28) | .02 | .11 |  |  |  |  | .916 |
|  | Smoking during preg | -0.10 (1.43) | -.01 | -.07 |  |  |  |  | .945 |
|  | Familial risk | -0.33 (0.27) | -.19 | -1.19 |  |  |  |  | .240 |
|  | Pubertal status | -1.87 (0.99) | -.33 | -1.90 |  |  |  |  | .064 |
|  | ETS exposure | 0.16 (0.07) | .31 | 2.12 |  |  |  |  | .040 |

*Notes. N* = 50. Dependent variable is the difference score in N2 amplitude between Nogo and Go trials, averaged over electrodes.

Table S36*. Go/NoGo: hierarchical regression model P3 NoGo minus Go*

| Predictor | | *B (Std. Error)* | *β* | *t* | *dfs* | *F* | *R^2^* | *∆R^2^* | *p* |
| --- | --- | --- | --- | --- | --- | --- | --- | --- | --- |
| Step 1 | |  |  |  | 4, 45 | 1.18 | .10 | .10 | .334 |
|  | Constant | 19.81 (4.58) |  | 4.32 |  |  |  |  | < .001 |
|  | Gender | 1.65 (2.14) | .14 | 0.77 |  |  |  |  | .443 |
|  | Smoking during preg | 2.68 (2.38) | .18 | 1.13 |  |  |  |  | .266 |
|  | Familial risk | -0.42 (0.46) | -.15 | -0.92 |  |  |  |  | .364 |
|  | Pubertal status | -3.23 (1.65) | -.35 | -1.96 |  |  |  |  | .056 |
| Step 2 | |  |  |  | 5, 44 | 0.97 | .32 | .01 | .444 |
|  | Constant | 19.87 (4.62) |  | 4.30 |  |  |  |  | < .001 |
|  | Gender | 1.75 (2.17) | .15 | 0.81 |  |  |  |  | .425 |
|  | Smoking during preg | 2.84 (2.42) | .19 | 1.17 |  |  |  |  | .247 |
|  | Familial risk | -0.39 (0.47) | -.14 | -0.84 |  |  |  |  | .406 |
|  | Pubertal status | -3.13 (1.67) | -.34 | -1.34 |  |  |  |  | .069 |
|  | ETS exposure | -0.06 (0.12) | -.08 | -0.50 |  |  |  |  | .621 |

*Notes. N* = 50. Dependent variable is the difference score in P3 amplitudes between NoGo and IfGo trials, averaged over electrodes.

Table S37*. Go/NoGo task: Separate regression models, difference score NoGo minus Go for N2 and P3 components, excluding covariates*

| Predictor | | *B (Std. Error)* | *β* | *t* | *dfs* | *F* | *R^2^* | *p* |
| --- | --- | --- | --- | --- | --- | --- | --- | --- |
| N2 diff. NoGo – Go^a^ | |  |  |  | 1, 49 | 1.79 | .04 | .187 |
|  | Constant | -4.06 (0.77) |  | -5.25 |  |  |  | < .001 |
|  | ETS exposure | 0.09 (0.09) | .19 | 1.34 |  |  |  | .187 |
| P3 diff. NoGo – Go^b^ | |  |  |  | 1, 49 | 0.65 | .12 | .423 |
|  | Constant | 11.46 (1.28) |  | 8.93 |  |  |  | < .001 |
|  | ETS exposure | -0.09 (0.12) | -.12 | -0.81 |  |  |  | .423 |

*Notes.* N = 51. In each model, the only predictor was the continuous measure of ETS exposure.

^a.^Dependent variable is the difference score in N2 amplitude between NoGo and Go trials. ^b.^Dependent variable is the difference score in P3 amplitude between NoGo and Go trials.

Difference scores were averaged over electrodes.

**Correlation matrices**

Table S38. *Correlations between predictors*

|  | 1 | 2 | 3 | 4 | 5 | 6 |
| --- | --- | --- | --- | --- | --- | --- |
| 1. ETS exposure dichotomous | - | - | - | - | - | - |
| 2. ETS exposure continuous | .87**(84) | - | .16 (52) | .22 (52) | .23 (51) | .32** (51) |
| 3. Gender | -.19 (84) | -.10 (84) | - | .56**(52) | .25 (51) | .11 (51) |
| 4. Pubertal status | .11 (84) | .20 (84) | .45**(84) | - | .04 (51) | .16 (51) |
| 5. Familial risk | .41**(82) | .33**(82) | .05 (82) | .01 (82) | - | .52** (51) |
| 6. Smoking during pregnancy | .20 (82) | .32**(82) | .07 (82) | .09 (82) | .53**(82) | - |

*Notes.* * *p* <.05 ** *p* < .01 *** *p* < .001 (two-tailed). Below the diagonal, data of all participants were included (*N* = 84), above the diagonal, only ETS exposed individuals were included (*N* = 52). Data for familial risk and smoking during pregnancy was missing for two participants, one of which was included in the ETS exposed group. Therefore, *n* = 82 below and *n* = 51 above the diagonal for cells involving those variables. Spearman’s rho was used for correlations involving dichotomous ETS exposure, Gender, and Smoking during pregnancy. Pearson correlations were used for all other correlations between two continuous variables.

Table S39*. Correlations between predictors and difference scores for the cue reactivity, Go/IfGo/NoGo, and Monetary Incentive Delay tasks, for the complete sample and ETS exposed group*

|  | **ETS exposure dichotomous** | | **ETS exposure continuous** | **Gender** | **Pubertal Status** | **Familial Risk** | **Smoking during pregnancy** |
| --- | --- | --- | --- | --- | --- | --- | --- |
|  | | **Full sample - Cue Reactivity task** | | | | | |
| P3 smoke – neutral | | -.04 (81) | -.10 (81) | .04 (81) | -.14 (81) | .01 (79) | -.13 (79) |
| P3 romantic – neutral | | .07 (81) | -.02 (81) | -.05 (81) | -.20 (81) | .12 (79) | -.05 (79) |
| LPP smoke – neutral | | -.09 (81) | -.01 (81) | .00 (81) | -.17 (81) | .00 (79) | -.06 (79) |
| LPP romantic – neutral | | -.11 (81) | -.10 (81) | .05 (81) | -.27* (81) | .12 (79) | .08 (79) |
|  | | **Full sample - Go/IfGo/NoGo task** | | | | | |
| Accuracy NoGo trials | | .14 (84) | .12 (84) | .31** (84) | .05 (84) | -.03 (82) | -.01 (82) |
| N2 NoGo – Go | | .02 (83) | .16 (83) | .01 (84) | -.13 (83) | -.09 (81) | .01 (81) |
| P3 NoGo – Go | | -.02 (83) | -.07 (83) | .01 (83) | -.19 (83) | .04 (81) | .17 (81) |
|  | | **Full sample - Monetary Incentive Delay task** | | | | | |
| Reaction times Reward – Non-reward | | .03 (84) | -.11 (84) | -.21 (85) | -.04 (84) | .02 (82) | .10 (82) |
| P3 Reward – Non-reward | | .02 (82) | -.17 (82) | -.07 (82) | -.21 (82) | -.01 (80) | -.12 (80) |
| FRN Reward_incorrect_ – Reward_correct_ | | -.04 (82) | .02 (77) | -.27* (77) | -.20 (77) | -.05 (75) | .14 (75) |
|  | | **ETS exposed group - Cue Reactivity task** | | | | | |
| P3 difference smoke- neutral | | - | -.10 (49) | -.14 (49) | -.19 (49) | -.01 (48) | -.29* (48) |
| P3 differerence romantic-neutral | | - | -.07 (49) | -.14 (49) | -.11 (49) | .14 (48) | -.24 (48) |
| LPP difference smoke-neutral | | - | .05 (49) | -.01 (49) | -.16 (49) | .06 (48) | -.09 (48) |
| LPP difference romantic-neutral | | - | -.05 (49) | .05 (49) | -.16 (49) | .23 (48) | .02 (48) |
|  | | **ETS exposed group - Go/IfGo/NoGo task** | | | | | |
| Behavioral NoGo accuracy | | - | .10 (51) | .38** (52) | .07 (52) | -.10 (51) | -.10 (51) |
| N2 diff. NoGo – Go | | - | .19 (52) | -.18 (51) | -.25 (51) | -.14 (50) | -.01 (50) |
| P3 diff. NoGo – Go | | - | -.12 (51) | -.20 (51) | 0.23 (51) | -.06 (50) | .04 (50) |
|  | | **ETS exposed group - Monetary Incentive Delay task** | | | | | |
| Reaction times Reward – Non-reward | | - | -.19 (52) | -.25 (52) | -.10 (52) | .03 (51) | .16 (51) |
| P3 Reward – Non-reward | | - | -.29* (50) | -.17 (50) | -.30* (50) | -.21 (49) | -.20 (49) |
| FRN Reward_incorrect_ – Reward_correct_ | | - | .04 (47) | -.48** (47) | -.21 (47) | -.16 (46) | .10 (46) |

*Notes.* * *p* <.05 ** *p* < .01 *** *p* < .001. Spearman’s rho was used for correlations involving dichotomous ETS exposure, Gender, and Smoking during pregnancy. Pearson correlations were used for all other correlations involving two continuous variables. The sample size for each correlation is given between brackets.

Table S40*. Correlations between valence and arousal ratings, P3 and LPP components and the ETS exposure measure for the complete sample and the ETS exposed group*

|  | **Valence Neutral** | | **Valence Smoke** | **Valence Romantic** | **Arousal Neutral** | **Arousal Smoke** | **Arousal Romantic** | **ETS exposure continious** |
| --- | --- | --- | --- | --- | --- | --- | --- | --- |
|  | | **Full sample** | | | | | |  |
| P3 neutral | | .126 (81) | -.021(81) | .149 (81) | -.026 (81) | -.016 (81) | -.027 (81) | .041 (81) |
| P3 smoke | | .097 (81) | -.015 (81) | .074 (81) | .011 (81) | .013 (81) | -.049 (81) | -.002 (81) |
| P3 romantic | | .091 (81) | -.056 (81) | .033 (81) | .065 (81) | .091 (81) | -.084 (81) | .027 (81) |
| LPP neutral | | .149 (81) | -.003 (81) | .112 (81) | .031 (81) | -.071 (81) | -.080 (81) | .097 (81) |
| LPP smoke | | .070 (81) | -.050 (81) | .022 (81) | .057 (81) | .055 (81) | -.104(81) | .082 (81) |
| LPP romantic | | .099 (81) | -.036 (81) | -.028 (81) | 0.134 (81) | .104 (81) | -.128 (81) | .022 (81) |
| ETS exposure continious | | -.029 (84) | .234*(84) | -.011 (84) | -.085 (84) | .029 (84) | -.018 (84) |  |
|  | | **ETS exposed group** | | | | | |  |
| P3 neutral | | .133 (49) | -.007 (49) | .164 (49) | -.146 (49) | -.099 (49) | .062 (49) | .110 (49) |
| P3 smoke | | .124 (49) | -.010 (49) | .122 (49) | -.125 (49) | -.080 (49) | .065 (49) | .066 (49) |
| P3 romantic | | .150 (49) | -.006 (49) | -.006 (49) | -.053 (49) | -.012 (49) | .039 (49) | .072 (49) |
| LPP neutral | | .189 (49) | .087 (49) | .168 (49) | -.011 (49) | -.158 (49) | .051 (49) | .077 (49) |
| LPP smoke | | .083 (49) | -.036 (49) | .080 (49) | -.069 (49) | -.114 (49) | .087 (49) | .111 (52) |
| LPP romantic | | .129 (49) | -.101 (49) | -.007 (49) | .047 (49) | -0.28 (49) | -.023 (49) | .042 (49) |
| ETS exposure continious | | .045 (52) | .271 (52) | .086 (52) | -.019 (52) | .016 (52) | .111 (52) |  |

*Notes.* * *p* <.05 . Pearson correlations were used for all correlations (involving two continuous variables). The sample size for each correlation is given between brackets.

**References Supplement**

Broyd, S. J., Richards, H. J., Helps, S. K., Chronaki, G., Bamford, S., & Sonuga-Barke, E. J. S. (2012). An electrophysiological monetary incentive delay (e-MID) task: A way to decompose the different components of neural response to positive and negative monetary reinforcement. *Journal of Neuroscience Methods*. https://doi.org/10.1016/j.jneumeth.2012.05.015

Glazer, J. E., Kelley, N. J., Pornpattananangkul, N., Mittal, V. A., & Nusslock, R. (2018). Beyond the FRN: Broadening the time-course of EEG and ERP components implicated in reward processing. *International Journal of Psychophysiology*. https://doi.org/10.1016/j.ijpsycho.2018.02.002

Goldstein, R. Z., Cottone, L. A., Jia, Z., Maloney, T., Volkow, N. D., & Squires, N. K. (2006). The effect of graded monetary reward on cognitive event-related potentials and behavior in young healthy adults. *International Journal of Psychophysiology*. https://doi.org/10.1016/j.ijpsycho.2006.05.006

Heatherton, T., Kozlowski, L., Frecker, R., & Fagerström, K. (1991). The Fagerström Test for Nicotine Dependence: a revision of the Fagerström Tolerance Questionnaire. - PubMed - NCBI. Retrieved from https://www.ncbi.nlm.nih.gov/pubmed/1932883

Johnson, R., & Donchin, E. (1980). P300 and Stimulus Categorization: Two Plus One is not so Different from One Plus One. *Psychophysiology*, *17*(2), 167–178. https://doi.org/10.1111/j.1469-8986.1980.tb00131.x

Luijten, M., Littel, M., & Franken, I. H. A. (2011). Deficits in inhibitory control in smokers during a Go/Nogo task: An investigation using event-related brain potentials. *PLoS ONE*. https://doi.org/10.1371/journal.pone.0018898

Marco-Pallares, J., Cucurell, D., Münte, T. F., Strien, N., & Rodriguez-Fornells, A. (2011). On the number of trials needed for a stable feedback-related negativity. *Psychophysiology*, *48*(6), 852–860. https://doi.org/10.1111/j.1469-8986.2010.01152.x

Petersen, A. C., Crockett, L., Richards, M., & Boxer, A. (1988). A self-report measure of pubertal status: Reliability, validity, and initial norms. *Journal of Youth and Adolescence*, *17*(2), 117–133. https://doi.org/10.1007/BF01537962

Pfabigan, D. M., Seidel, E. M., Sladky, R., Hahn, A., Paul, K., Grahl, A., … Lamm, C. (2014). P300 amplitude variation is related to ventral striatum BOLD response during gain and loss anticipation: An EEG and fMRI experiment. *NeuroImage*. https://doi.org/10.1016/j.neuroimage.2014.03.077

Piasecki, T. M., Fleming, K. A., Trela, C. J., & Bartholow, B. D. (2017). P3 event-related potential reactivity to smoking cues: Relations with craving, tobacco dependence, and alcohol sensitivity in young adult smokers. In *Psychology of Addictive Behaviors*. https://doi.org/10.1037/adb0000233

Pornpattananangkul, N., & Nusslock, R. (2015). Motivated to win: Relationship between anticipatory and outcome reward-related neural activity. *Brain and Cognition*. https://doi.org/10.1016/j.bandc.2015.09.002

Vink, J. M., Willemsen, G., Beem, A. L., & Boomsma, D. I. (2005). The Fagerström Test for Nicotine Dependence in a Dutch sample of daily smokers and ex-smokers. *Addictive Behaviors*, *30*(3), 575–579. https://doi.org/10.1016/j.addbeh.2004.05.023

Vink, J. M., Willemsen, G., & Boomsma, D. I. (2005). Heritability of Smoking Initiation and Nicotine Dependence. *Behavior Genetics*, *35*(4), 397–406. https://doi.org/10.1007/s10519-004-1327-8
