# Supplementary figures and images for: Effects of environmental tobacco smoke exposure on brain functioning in never‐smoking adolescents
Source: Brain Behav. 2020 Jul 1;10(8):e01619. doi: 10.1002/brb3.1619 (PMC7428475; doi:10.1002/brb3.1619)

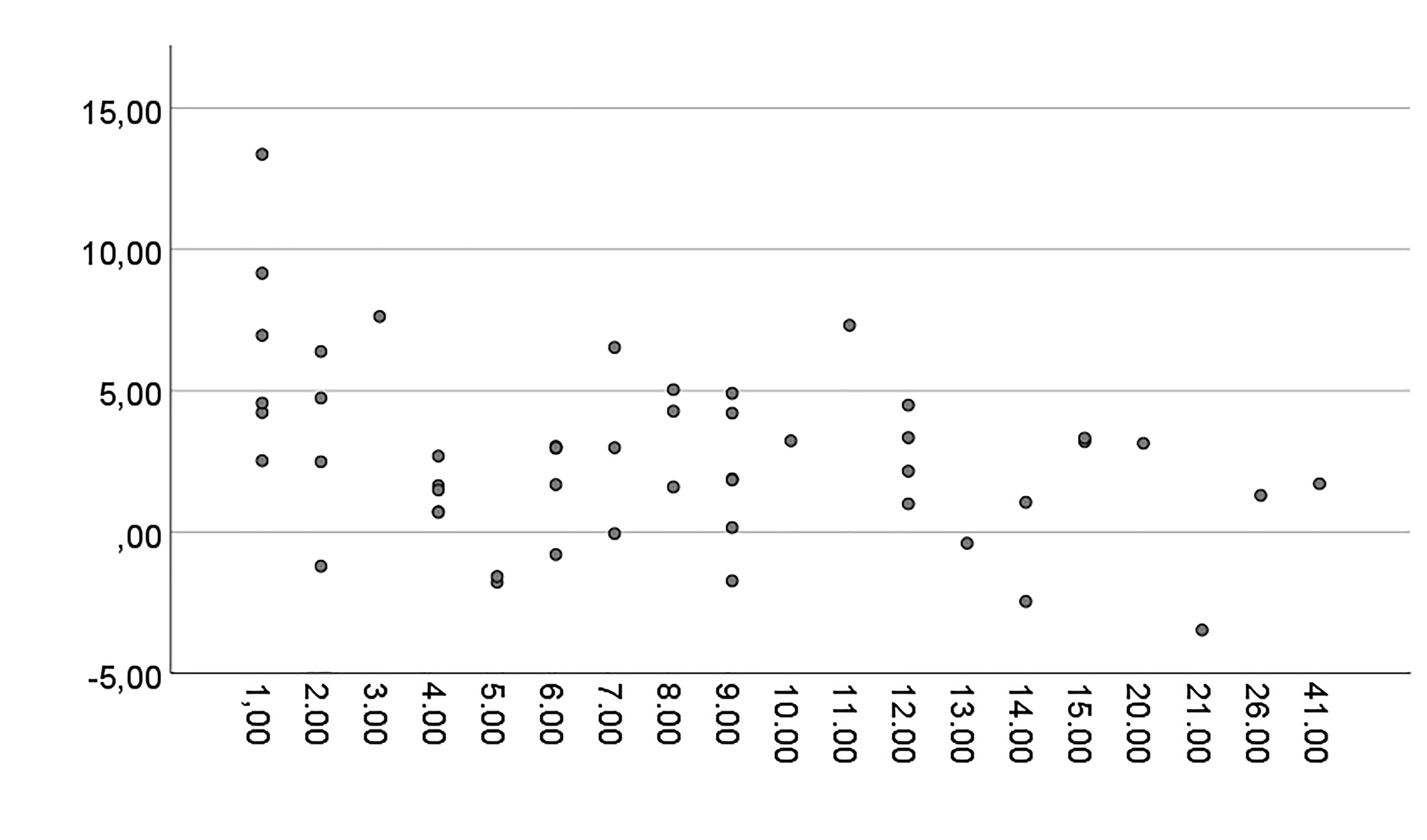

Supplement: Supplementary file 2 — Fig S1 [file BRB3-10-e01619-s002.tif]
